# Genetic correlation and causal relationships between cardio-metabolic traits and lung function impairment

**DOI:** 10.1186/s13073-021-00914-x

**Published:** 2021-06-21

**Authors:** Matthias Wielscher, Andre F. S. Amaral, Diana van der Plaat, Louise V. Wain, Sylvain Sebert, David Mosen-Ansorena, Juha Auvinen, Karl-Heinz Herzig, Abbas Dehghan, Debbie L. Jarvis, Marjo-Riitta Jarvelin

**Affiliations:** 1 Department of Epidemiology and Biostatistics, MRC-PHE Centre for Environment and Health, School of Public Health, Imperial College London, Norfolk Place, London, W2 1PG UK; 2 National Heart and Lung Institute (NHLI), Imperial College London, Emmanuel Kaye Building, London, SW3 6LR UK; 3 Genetic Epidemiology Group, Department of Health Sciences, George Davies Centre, University of Leicester, University Rd, Leicester, LE1 7RH UK; 4 National Institute for Health Research, Leicester Respiratory Biomedical Research Centre, Glenfield Hospital, University Rd, Leicester, LE1 7RH UK; 5 Center for Life Course Health Research, Faculty of Medicine, University of Oulu, P.O.Box 8000, FI-90014 Oulu, Finland; 6 Biocenter of Oulu, University of Oulu, Aapistie 5, FI-90014 Oulu, Finland; 7 Research Unit of Biomedicine, Medical Research Center (MRC), University of Oulu, University Hospital, P.O. Box 8000, Oulu, Finland; 8 Department of Gastroenterology and Metabolism, Poznan University of Medical Sciences, 41 Jackowskiego St, 60-512 Poznan, Poland; 9 Department of Life Sciences, College of Health and Life Sciences, Brunel University London, Kingston Lane, London, UB8 3PH UK

**Keywords:** Mendelian randomisation, Metabolic syndrome, Obesity, Chronic obstructive pulmonary disease, UK Biobank

## Abstract

**Background:**

Associations of low lung function with features of poor cardio-metabolic health have been reported. It is, however, unclear whether these co-morbidities reflect causal associations, shared genetic heritability or are confounded by environmental factors.

**Methods:**

We performed three analyses: (1) cardio-metabolic health to lung function association tests in Northern Finland Birth cohort 1966, (2) cross-trait linkage disequilibrium score regression (LDSC) to compare genetic backgrounds and (3) Mendelian randomisation (MR) analysis to assess the causal effect of cardio-metabolic traits and disease on lung function, and vice versa (bidirectional MR). Genetic associations were obtained from the UK Biobank data or published large-scale genome-wide association studies (*N* > 82,000).

**Results:**

We observed a negative genetic correlation between lung function and cardio-metabolic traits and diseases. In Mendelian Randomisation analysis (MR), we found associations between type 2 diabetes (T2D) instruments and forced vital capacity (FVC) as well as FEV1/FVC. Body mass index (BMI) instruments were associated to all lung function traits and C-reactive protein (CRP) instruments to FVC. These genetic associations provide evidence for a causal effect of cardio-metabolic traits on lung function. Multivariable MR suggested independence of these causal effects from other tested cardio-metabolic traits and diseases. Analysis of lung function specific SNPs revealed a potential causal effect of FEV1/FVC on blood pressure.

**Conclusions:**

The present study overcomes many limitations of observational studies by using Mendelian Randomisation. We provide evidence for an independent causal effect of T2D, CRP and BMI on lung function with some of the T2D effect on lung function being attributed to inflammatory mechanisms. Furthermore, this analysis suggests a potential causal effect of FEV1/FVC on blood pressure. Our detailed analysis of the interplay between cardio-metabolic traits and impaired lung function provides the opportunity to improve the quality of existing intervention strategies.

**Supplementary Information:**

The online version contains supplementary material available at 10.1186/s13073-021-00914-x.

## Background

Obesity and cardio-metabolic traits have become an increasing public health problem in most parts of the world. By 2025, global obesity prevalence is predicted to reach 18% in men and 21% in women [1]. The associations of obesity with chronic non-communicable diseases such as type 2 diabetes, cardiovascular disease and cancers are well described. Meanwhile, there is a growing literature on the association of obesity with lung function and chronic lung disease, although the underlying pathways and potential mediators are not well understood.

Several observational studies have reported an association between low lung function and cardio-metabolic traits, including obesity [2–5]. In this report, we replicated these associations using data from a population-based cohort, the Northern Finland Birth Cohort (NFBC1966). However, it is not possible to infer whether associations such as those seen in NFBC1966, and in the other studies, are causal as most studies were not able to control for all known potential confounders or residual confounding by unknown factors.

**Fig. 1:**
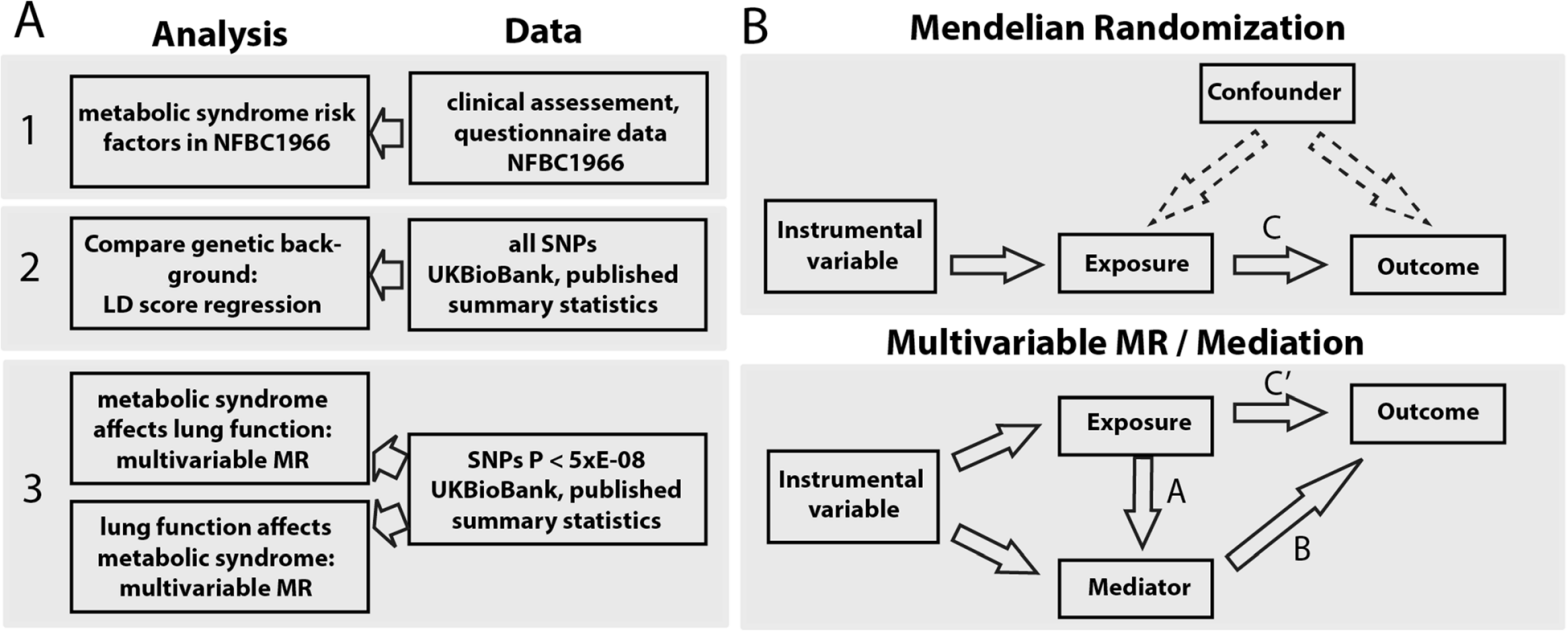
Flow chart of study design. **A** Cardio-metabolic traits were body mass index (BMI), type 2 diabetes (T2D), C-reactive protein (CRP), lipoprotein (HDL-C), low-density lipoprotein (LDL-C), total cholesterol (TC), triglycerides (TG), systolic blood pressure (SBP), diastolic blood pressure (DBP), pulse pressure (PP) and coronary artery disease (CAD). Tested lung function traits were first second forced expiratory capacity (FEV1), forced vital capacity (FVC) and a ratio of both FEV1/FVC. **B** Graphical relationship diagrams in a classical MR and mediation analysis. Upper panel gives overview of MR analysis, indicating the use of genetic instruments instead of the actual exposure. Arrow labelled with C in upper panel refers to the causal estimate as well as the C-path in mediation analysis setting. Lower panel gives an overview of the mediation analysis following Baron-Kenney approach. For mediation analysis in this study, we subtracted C’ path from C-path to get effect sizes for mediation

In this study, we assess associations between eleven cardio-metabolic traits representing wider range of traits than usually accounted in pure metabolic syndrome definition [6, 7] body mass index (BMI) [8], type 2 diabetes (T2D) [9], C-reactive protein (CRP) [10], high-density lipoprotein (HDL), low-density lipoprotein (LDL), total cholesterol (TC), triglycerides (TG) [11], diastolic blood pressure (DBP), systolic blood pressure (SBP), pulse pressure (PP) [12], coronary artery disease (CAD) [13], and three lung function outcomes (first second forced expiratory volume (FEV1), forced vital capacity (FVC) and a ratio of both FEV1/FVC). We examine whether these metabolic traits and lung function are genetically correlated using a cross-trait linkage disequilibrium (LD) score regression and then go on to determine whether the associations are likely to be causal, using Mendelian Randomisation (MR). MR is a method to estimate causal effects by using genetic variants with known effects on the risk factor of interest as a proxy (i.e. instrumental variable) [14] and, as long as underlying assumptions are not violated [15, 16], MR is not susceptible to classical confounding (as seen in observational studies) or reverse causation (Fig. 1a).

Mendelian randomisation has been used to estimate the causal effect of body mass index (BMI) on two lung function parameters (forced expiratory volume in 1 s, FEV1, and forced vital capacity, FVC) [17]. However, it did not account for potential shared genetic instruments (in this case whether the genetic instruments modify lung function through factors other than BMI). To overcome this problem, we used a wide range of MR methods, amongst others a recently developed extension of MR, multivariable MR (MMR) [18], which models the effects of pleiotropy, estimating the independent causal effects of each risk factor simultaneously. This analysis setting allowed us to take advantage of horizontal pleiotropy and to gain insights into the interplay between cardio-metabolic traits through the comparison of MMR estimates and univariable MR estimates of each risk factor (mediation analysis). Moreover, former papers did not test for potential causal effects in the opposite direction as we do here by conducting a bidirectional MR (Fig. 1B).

## Methods

### Studied traits

In this study, we assessed associations between eleven cardio-metabolic traits and three lung function outcomes forced expiratory volume in 1 s (FEV1), forced vital capacity (FVC) and the ratio (FEV1/FVC) [19] (Table 1). Cardio-metabolic traits are body mass index (BMI) [8], type 2 diabetes (T2D) [9], C-reactive protein (CRP) [10], four blood lipid levels outcomes (high-density lipoprotein (HDL-C), low-density lipoprotein (LDL-C), total cholesterol (TC), and triglycerides (TG) [11], three blood pressure outcomes (diastolic blood pressure (DBP), systolic blood pressure (SBP), and pulse pressure (PP)) [12], coronary artery disease (CAD) [13], and three lung function outcomes (FEV1, FVC and the ratio of FEV1/FVC). Sensitivity analysis was preformed using data from Liu et al. for alcohol and tobacco addiction [20], alongside with Wood et al. [21] and Shungin et al. [22] for height and waist to hip ratio.

**Table 1:** Data used for the Mendelian randomization analysis. For CAD and T2D participant numbers were split into cases and controls. *Reproducibility of spirometry measurement using ERS/ATS criteria; **European ancestry; ***Stage 1 meta-analysis

Trait	Source	Year	Imputation panel	N	Trait transformation
**FEV1**	UKBB & Wain et al. [19]	2017	HRC	270381*	Raw, in liter
**FVC**	UKBB & Wain et al. [19]	2017	HRC	270381*	Raw, in liter
**FEV1/FVC**	UKBB & Wain et al. [19]	2017	HRC	270381*	Raw, in liter
**BMI**	Locke et al. [8]	2015	HapMap2	322154**	Rank inverse normal transformed (BMI~age + age^2 + sex)
**T2D**	Scott et al. [9]	2017	1kG	26676 (132532)	Case control
**CRP**	Dehghan et al. [10]	2011	HapMap2	82725	ln(hsCRP)
**HDL**	Willer et al. [11]	2013	HapMap2	188577	Rank inverse normal transformation (HDL~age+age2+sex)
**LDL**	Willer et al. [11]	2013	HapMap2	188577	Rank inverse normal transformation (LDL~age+age2+sex)
**TC**	Willer et al. [11]	2013	HapMap2	188577	Rank inverse normal transformation (TC~age+age2+sex)
**TG**	Willer et al. [11]	2013	HapMap2	188577	Rank inverse normal transformation (TG~age+age2+sex)
**SBP**	Wain et al. [12]	2017	1kG	150134***	Residuals of (SBP~sex + age + age^2 + BMI)
**DBP**	Wain et al. [12]	2017	1kG	150134***	Residulas of (DBP~sex + age + age^2 + BMI)
**PP**	Wain et al. [12]	2017	1kG	150134***	Residuals of (PP~sex + age + age^2 + BMI)
**CAD**	Nikpay et al. [13]	2015	1kG	60801 (123504)	Case control

### Observational associations with lung function in NFBC1966

The Northern Finland Birth Cohort 1966 (NFBC1966), described in detail previously [23, 24], targeted all pregnant women, residing in the two northernmost provinces of Finland with expected dates of delivery between January 1 and December 31, 1966. Over 96% of eligible women participated in the study, giving birth to 12,058 live born children. In 2012, at offspring age of 46 years, all cohort participants with known addresses and living in Northern Finland or Helsinki area were invited to a clinical examination, which included blood sampling. Clinical data and blood was collected from 5,861 participants. Lung function was assessed with a Vitalograph P spirometer (Vitalograph Ltd., Maids Moreton, UK). The present analysis is based on the best (highest) available lung function measure from participants who performed at least three acceptable blows, with the difference between two maximal readings of FEV1 or FVC less than 4% [25]. Associations of lung function (FEV1, FVC, FEV1/FVC) with cardio-metabolic traits were investigated in linear regression models adjusted for sex (male/female), age (years), height (cm), smoking status (current, former and never smokers) and pack-years (Additional file 1, Table S1).

Type 2 diabetes in NFBC1966 was defined as either prescription of metformin (Finnish register for reimbursed medication; ATC code A10B, available from year 1997 and 2016), diagnosed by a physician (Finnish outpatient register; ACD9 or 10), or screen-detected by OGTT at the age of 46 years (NFBC1966 clinical follow-up in 2012). Coronary heart disease was defined based on participants answer to the following questions: ‘Do you now or have you had following the doctor diagnosed or treated the symptoms, diseases or injuries: Congenital heart disease’.

For spirometry measurements, we used a MasterScreen Pneumo Spirometer (Vitalograph Ltd., Buckingham, UK), with a volumetric accuracy of ±2% or ±50 mL whichever was greater. The machines were calibrated every day the medical examination took place. The spirometric manoeuvre was performed three times in an upright sitting position while wearing a nose clip, but repeated if the coefficient of variation between two maximal readings was >4%.

### Associations of SNPs with cardio-metabolic traits

We extracted the effect estimates for SNPs associated (P< 5×10^-8^) with the cardio-metabolic traits from the most recent published GWAS including 82,000 up to 322,000 individuals (Table 1, Additional File 1: Table S2, Additional File 2: Table S3) [8–11, 13, 19]. We identified a set of non-overlapping independent variants for each risk factor via LD-pruning *r*^2^ < 0.2 within a window of 1MB using unrelated white European 1000 genomes v3 samples as reference. Clumping was performed using plink v1.9. An overview of LD correlation between exposure SNPs is given in table S4 in additional file 2.

### Associations of SNPs with lung function

We obtained effect estimates of the selected cardio-metabolic SNPs on lung function (FEV1, FVC and FEV1/FVC) from the UK Biobank (UKB; Application Number 19136) using BOLT-LMM adjusted for assessment centre, sex, age, height, current smoking status and pack-years (See Additional File 1: Table S5 for UKB characteristics).

### Cross-trait LD score regression

We assessed the genetic correlation between each metabolic trait and each lung function parameter using the recommended settings in the software package LDSC (v1.0.0) [26]. Briefly, this method generates a score reflecting whether the GWAS test statistic of a biologically relevant variant correlates with nearby variants in high linkage disequilibrium. The z statistic for the genetic association of each variant with trait 1 are multiplied with the z statistic for the genetic association with trait 2, followed by regression of this product of statistics against the LD scores. The slope (coefficient) represents genetic correlation. When large, the same genetic variants impact both the traits.

### Mendelian randomisation (MR)

We performed a 2-sample Mendelian randomisation, using the CRAN package *Mendelianrandomization*, unless stated otherwise (Fig. 1B). We excluded palindromic SNPs and instruments having a direct effect on the outcome (P<5×E−08). We estimated the causal effect of a single risk factor on lung function using the widely used fixed-effect inverse variance weighted (IVW) MR. We performed sensitivity analyses using weighted median, mode based and MR Egger methods to rule out potential pleiotropy. To further assess the stability of our results, we used penalised MR approaches and reproduced the results using altered sets on input SNPs. We achieved this via exclusion of critical variants as suggested by MR-PRESSO [27] and contamination mixture method [28] (Additional File 1: Supplementary methods, Additional File 2: Table S6). Throughout the paper, we present raw *P* values. The Bonferroni threshold correcting for 9 tests would be 5.5 × 10^-3^. Supplemental Figure S4 gives an overview of the correlation structure within cardiometabolic traits and a principal component analysis of 11 cardio-metabolic traits in NFBC1966. This analysis suggests that the first 3 principal components of the 11 cardio-metabolic explain 99% of the variance. Thus, we calculated, using a similar approach as metabolic profiling studies [29, 30], 3 outcomes (FEV1, FVC and FEV1/FVC) times 3 risk factors (first 3 principal components).

### Multivariable MR (MMR)

To assess the independent effects of each cardio-metabolic trait, while accounting for the effects of the others, we used multivariable MR [18] (Additional File 1: Supplementary methods, Additional File 2: Table S7). We regressed the coefficients for the SNP-outcome association against all risk factors separately and then simultaneously for each risk factor. The residuals of these regressions were used as the outcome to estimate the causal effect. We used a weighted regression-based approach to achieve this. To examine whether our findings were influenced by alcohol or tobacco use, height as well as waist to hip-related pleiotropic effects, we extended our analysis and included anthropometric traits in multivariable MR analysis (Additional File 1, Fig. S4).

### Effect attenuation and mediation analysis

Traits were interpreted as mediators when they were consistently significantly (*p*<0.05) associated with lung function in the univariable MR (B–path), and we could find evidence for a causal effect of the exposure on the mediator (A-path). These assumptions were fulfilled for the CRP-mediated effect of BMI on lung function. For this trait, we compared the direct effect estimates (IVW MR of risk factor) with the total effect estimate (MMR estimate of risk factor plus mediator) [31](Fig. 1B). Differences in effect sizes caused by all other traits were interpreted as effect attenuation.

### Bidirectional MMR

We repeated the MR analysis in the opposite direction to determine possible causal effects of lung function on cardio-metabolic traits (Fig. 1B). For this, we used a set of validated SNPs described by Wain et al. [19] as instruments for lung function (Additional File 1: Supplementary methods, Additional File 1: Table S5).

## Results

Cardio-metabolic traits are closely related and correlation across traits could generate horizontal pleiotropy. For example, if an instrument for trait A is also associated with trait B (e.g. variants in FTO for BMI and CRP), it would be challenging to find out whether the association with outcome (e.g. lung function) is reflecting the causal effects of traits A or B. This is a major challenge in this study as 17% of the variants we used as instruments in this MR are associated with more than one cardio-metabolic trait (Additional File 2: Table S3). To address this, we use outlier robust methods such as weighted median MR and mode-based estimation (Fig. 3), and also we performed sensitivity analysis on altered sets variants for each exposure (Additional File 1: Supplementary methods, Additional File 2: Table S6). Finally, by adding every risk factor to our MR model separately (MMR), we evaluate the independence of the tested effect as well as the attenuation as mediated fraction of the added risk factor on the outcome (Fig. 4).

**Fig. 2:**
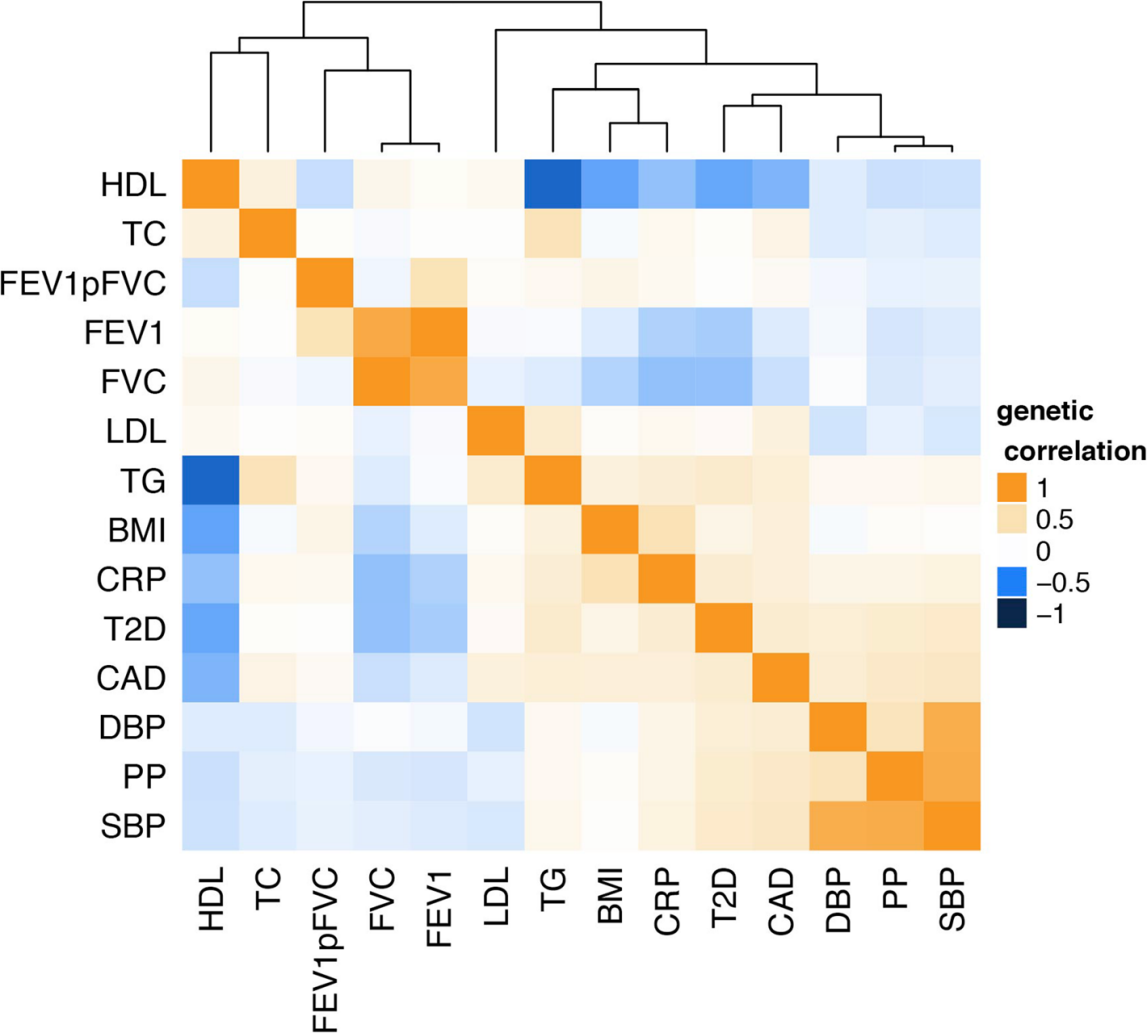
Heat map of genetic correlation (cross-trait LD score regression). Blue boxes indicate negative correlation; orange boxes positive genetic correlation. Distance on cluster dendrogramm measures the similarity between traits. Correlation values including *P* values between lung function traits and cardio-metabolic traits are given in table S9 in Additional File 1

**Fig. 3:**
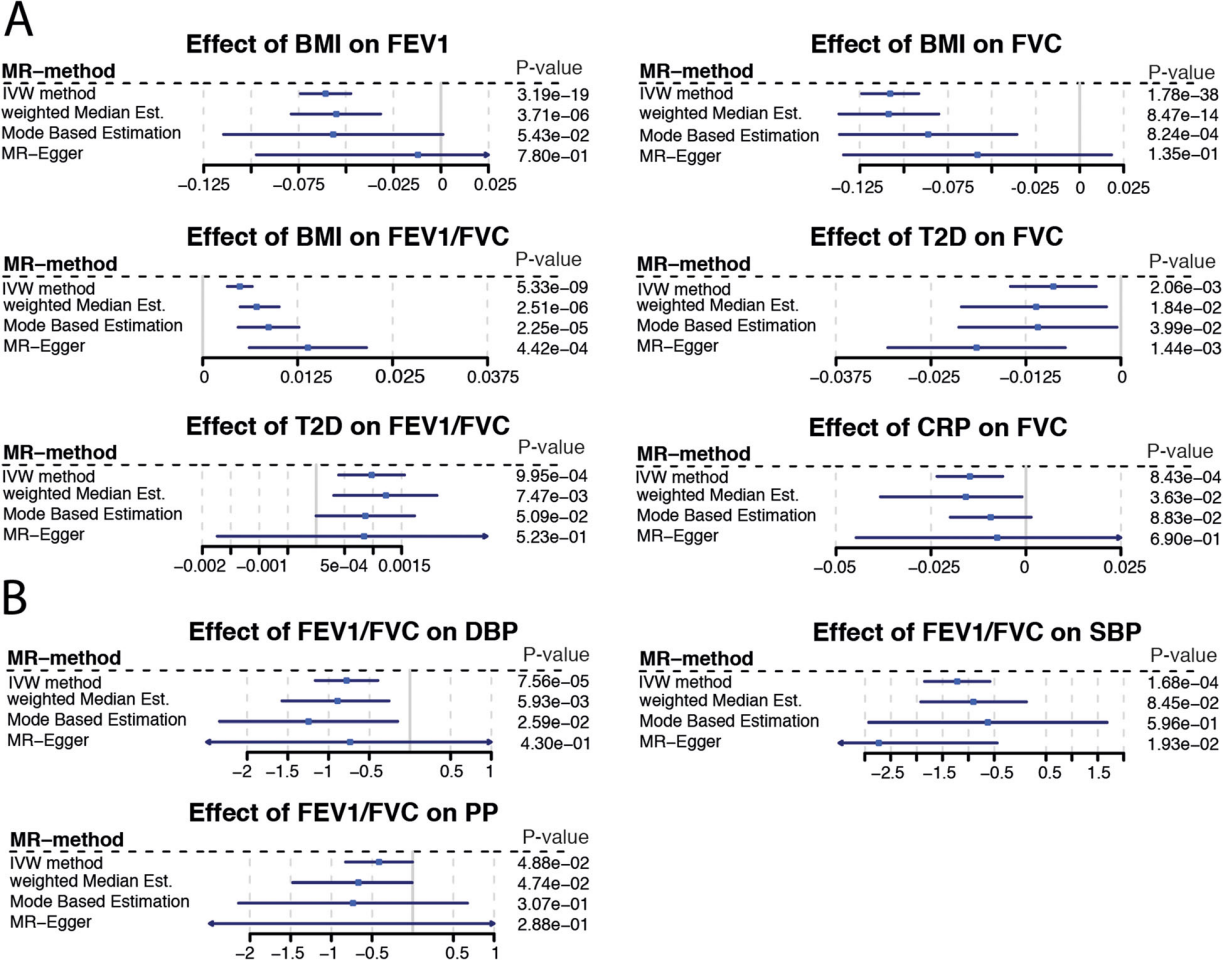
Forest plot of Mendelian randomisation result. Blue square represents causal estimate. Blue line is 95% confidence interval. Every line represents one approach to estimate the potential causal effect (Additional File 1: Supplementary methods). Section A represents effects of tested risk factors on impaired lung function. Section B is the inverse direction. Impaired lung function as exposure for blood pressure. If causal effect estimates were not nominal significant with at least two different approaches and did not have a consistent direction of effect they are given in Fig. S8-S13 in Additional File 1

**Fig. 4:**
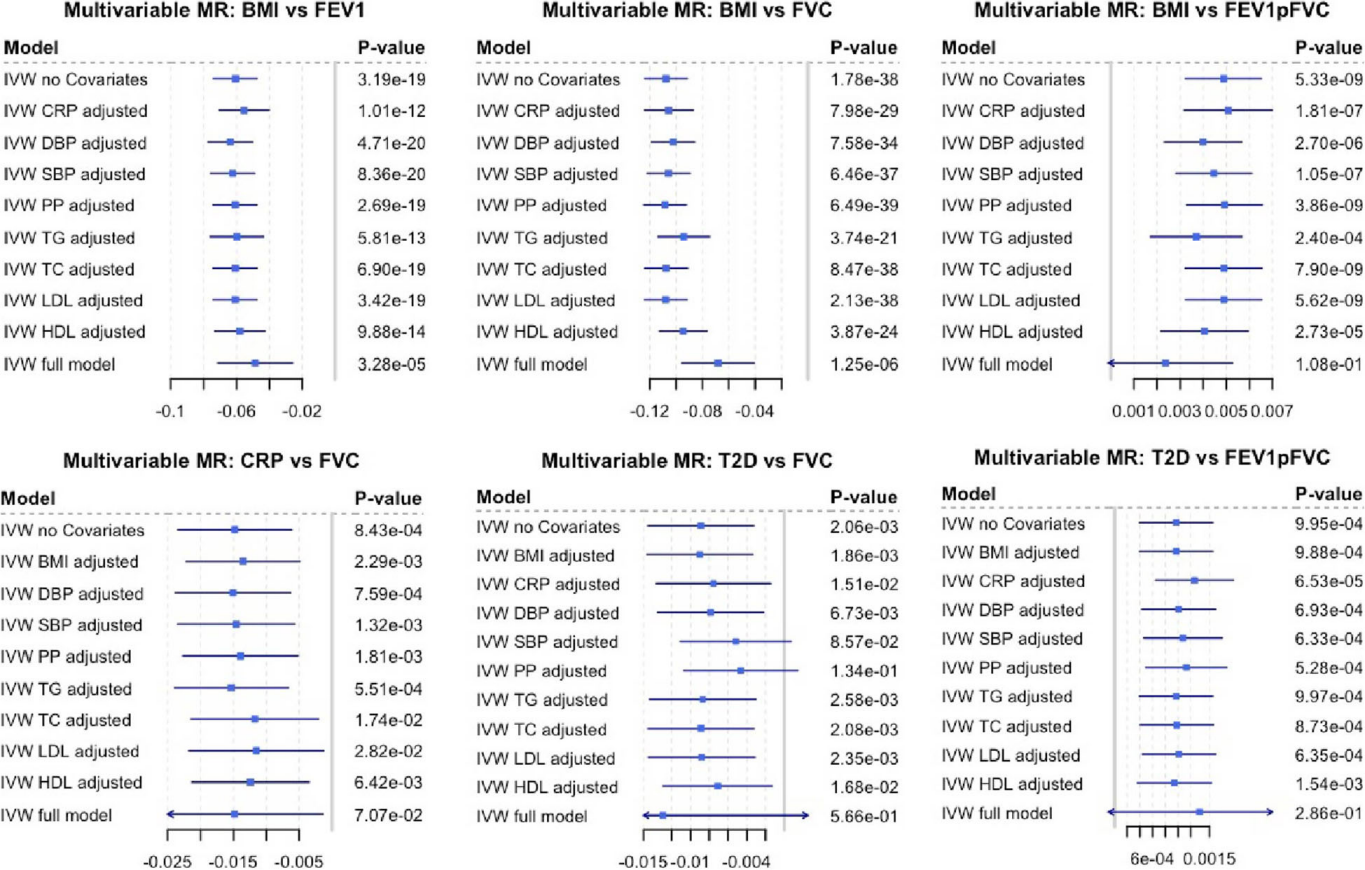
Multivariable MR and mediation analysis. First entry in each plot is the inverse variance-weighted causal estimate as given in Fig. 3. This estimate represents the direct effect of the risk factor on the outcome. Subsequent lines are adjusted for one risk factor each representing the total effect. Full model has all risk factors as covariate in the model. Differences in effect sizes resulting from attenuation can be interpreted as mediated by the exposure added to the model, if there is a causal connection between the mediator and the exposure as well as the mediator and the outcome

In the following, we present results of the three analyses for each cardio-metabolic trait (Fig. 1):

1. Observational analyses of the association between traits, using the data from the NFBC1966 study (Additional File 1: Table S1, S8).
2. Evidence for genetic correlation using cross-trait LD score regression (Fig. 2, Additional File 1: Table S9).
3. Evidence for causal associations from Mendelian randomisation analysis of cardio-metabolic traits on lung function, and vice-versa (Fig. 3, Additional File 1: Fig. S8-S13, Additional File 2: Table S6). Robust associations between instruments of the risk factors and outcomes were followed-up by multivariable MR (Fig. 4). This allowed us to draw conclusions if any potential causal effects were independent from other tested risk factors (horizontal pleiotropy) and if we can find certain proportions of the effect being mediated by other cardio-metabolic traits.

### BMI

We found negative associations of BMI with FEV1 and FVC, and positive associations with FEV1/FVC ratio in the observational analyses in NFBC1966. Consistent with this, cross-trait LD score regression showed the same direction of effects (FEV1/FVC P=1.8×E−13, FEV1 P=8×E−04, FVC P=9.9×E−18; Fig. 2). For Mendelian randomisation analysis, we used BMI-specific SNPs described in Locke et al. [8]. F-statistic of variants was 27 or higher (Additional File 2: Table S3). We note that 12 SNPs deployed as BMI instruments also reach genome-wide significance for one or more of the tested cardio-metabolic traits (Additional File 2: Table S3). Evaluation of several MR approaches suggests a causal effect of BMI on all lung function parameters (Fig. 3A). Effect sizes derived from the IVW MR analysis in the UK Biobank data showed a decrease of 24ml in FVC and 12ml of FEV1 per unit (kg/m^2^) change in BMI. Low *P* values obtained from multivariable MR (MMR) suggest that the effects of BMI on lung function are independent from other tested risk factors (Fig. 4). However, we observed some attenuation of BMI effects on FVC and FEV1/FVC when adding genetic instruments for diastolic blood pressure, triglycerides or HDL-C to multivariable MR model (Additional File 2: Table S10, Fig. 4). Similarly, we found some effect attenuation when adjusting the multivariable MR model for smoking and alcohol consumption. Other anthropometric traits such as height and waist to hip ratio had only small effects on BMI lung function associations (Additional File 1: Fig. S4). Multivariable MR also suggests that 2% of the BMI effect on restrictive lung patterns (indicated by lower FVC values) is mediated by CRP. We observed a doubling in effect sizes when comparing associations between BMI instruments and female FEV1 or FVC values to male lung function. There was no sex-specific difference in effect sizes for BMI FEV1/FVC association (Additional File 1 Fig. S14).

### Type 2 diabetes

As seen for BMI, T2D was negatively associated with FEV1 and FVC and positively associated with FEV1/FVC lung patterns in NFBC1966 (Additional File 1: Table S8). Cross-trait LD score regression showed a negative genetic correlation with both FVC (P=9.2×E−13) and FEV1 (P=3.1×E−10) and a positive but less pronounced genetic correlation with FEV1/FVC (P=0.015, Fig. 2). We used genetic instruments for T2D described in Scott et al. [9] (Table 1). All variants had an F statistic above 30 (Additional File 2: Table S3). Eleven T2D instruments were associated to one or more of the other tested risk factors in this study. Applying several MR techniques, we found a consistent association between T2D-specific SNPs and FVC and FEV1/FVC (Fig. 3). Effect sizes derived from the IVW MR analysis suggested a decrease of 65 ml in FVC and 108 ml of FEV1 with the presence of T2D. *P* values obtained from multivariable MR indicate the effect of T2D on impaired lung function is independent from most tested risk factors. We observed strong attenuation of the T2D effect on FVC when adding instruments for SBP or PP (Fig. 4) to multivariable regression model. Adding genetic instruments for CRP to the model shows 4.9% of the T2D effect on FVC can be attributed to CRP. We observed some effect attenuation of the T2D effect on FEV1/FVC when adding smoking as covariate (Additional File 1: Fig. S4). There was no significant effect attenuation when adding BMI or other anthropometric traits to the regression model (Fig. 4, Additional File 1: Fig. S4).

### CRP

Within NFBC, we found a strong negative association between blood CRP levels and all three lung function parameters (Additional File 1: Table S8). There was strong evidence of a genetic correlation between lung function and blood CRP levels (FEV1/FVC P=0.0528, FEV1 P=4.29E−06, FVC P=1.15E−11; Additional File 1: Table S9, Fig. 2). We used CRP instruments described in Dehghan et al. (Table 1) [10]. We note that seven out of the 18 CRP instruments were associated with one or more cardio-metabolic trait (Additional File 2: Table S3). Genetic instruments for CRP were significantly associated with FVC, suggesting a causal effect of CRP on restrictive lung patterns (Fig. 3). We observed a decrease of 14ml in FVC per log change in serum CRP level. MMR analysis supports a causal effect of CRP on FVC with strong attenuation of the effect when adding total cholesterol, LDL-C or HDL-C, CRP or smoking to the model (Fig. 4, Additional File 1: Fig. S4).

### Lipid levels

HDL cholesterol and triglyceride levels were associated with lung function in NFBC1966 (Fig. 3). There was a negative genetic correlation of triglycerides with FVC (*P*=0.029) but a positive correlation with FEV1/FVC (*P*=0.012), with the opposite for HDL cholesterol (positive correlation with FVC, P=3.8×E−03; negative correlation with FEV1/FVC, P=4.5×E−04, Additional File 1: Table S9). For LDL-C and total cholesterol, no significant associations were seen in the observational data nor was there evidence of genetic correlation. We did not see consistent associations between any lipid trait and lung function in the MR analyses (Additional File 1: Fig. S8-S13).

### Blood pressure

We observed a negative association of both DBP and SBP with FEV1 and FVC and a positive association with FEV1/FVC (Additional File 1: Table S8, Fig. 3) in NFBC1966, but no associations with PP. LD score regression showed negative genetic correlation between FEV1 and PP (P=1.8×E−03, Fig. 2, Additional File 1: Table S9) only. We did not observe consistent associations between any blood pressure trait and lung function in MR analyses (Additional File 1: Fig. S8-S10)

### Coronary artery disease

A diagnosis of CAD was not associated with lung function in NFBC1966 (Additional file 1: Table S8). Cross-trait LD score regression showed a negative correlation with FEV1 and FVC, but a positive correlation with FEV1/FVC (FEV1/FVC P=8.2×E−03, FEV1 P=2×E−03, FVC P=3.1×E−06, Fig. 2). MR suggested these correlations were not causal. (Additional File 1: Fig. S8-S10).

### Causal effect of lung function on cardio-metabolic traits

We used variants described by Wain et al. [19] as instruments (Table 1) to test for possible causal effects of lung function on cardio-metabolic traits. We discovered consistent associations between FEV1/FVC-specific SNPs and DBP, SBP as well as PP suggesting a causal effect of FEV1/FVC on blood pressure (Fig. 3B, Additional File 1: Fig. S10-S13).

## Discussion

In summary, our findings suggest that lung function parameters are genetically correlated with multiple cardio-metabolic traits (BMI, T2D, CRP, HDL-C, LDL-C, TC, TG, SBP, DBP, PP, CAD). Furthermore, we found evidence for causal effect of some cardio-metabolic traits (BMI, T2D, CRP) on lung function measures and a possible causal effect of FEV1/FVC on blood pressure. As the assessed cardio-metabolic traits were highly correlated, we used multivariable Mendelian randomisation (MMR) to validate the findings from univariable MR and investigate the interplay between cardio-metabolic traits as the method simultaneously accounts for multiple causal factors (Figs. 1B and 4).

We have used the state-of-the-art statistical methods for assessing genetic correlation, using publicly available summary statistics of genetic associations. This method has been previously used to assess genetic correlations for example between 24 traits (including cardio-metabolic traits, mental health disorders, inflammatory bowel disease and educational attainment but not respiratory conditions) [26], between thirteen growth and eleven immune phenotypes (including asthma) [32], and between six cancers (including lung) and 14 non-cancer diseases (not respiratory conditions) [33]. To our knowledge, this method has not been used to report genetic correlations between cardio-metabolic traits and lung function measures as shown here. One study has reported nominally significant genetic correlation with COPD for resting heart rate and hypertension (considered as a binary measure), with no correlation seen between COPD and stroke and other blood pressure traits [34].

We went on to determine whether the observed correlations could reflect causal associations using MR. LD score regression is not the same as Mendelian randomisation as it uses information from the whole genome and thus does not model one trait as a function of the other. Also, it makes no assumption of the causal direction of association (whereas MR tests the effect of one factor on the other). We show through MR that higher BMI is causally associated with lower FEV1 and FVC, with greater effects on the latter, explaining its positive effect on the derived parameter FEV1/FVC. This is consistent with previous reports from multiple large-scale population-based epidemiological studies [35], as well as, with observational results from our analyses in the NFBC1966. Our Mendelian randomisation confirms these associations are very unlikely to be related to confounding by lifestyle factors related to BMI and lung function measures. Some have hypothesised that these associations could reflect reverse causation e.g. people with reduced lung function may be less likely to engage into physically activity and may subsequently gain weight. However, our bidirectional MR did not support this hypothesis.

### Body mass index

Our analysis showed stronger effects of increased BMI on restrictive ventilation patterns than on airway obstruction. The mechanisms for how BMI affects lung function remain elusive although it is likely that fat accumulation between the muscles around the lungs and in the abdomen may have mechanical effects on the diaphragm and impede full inspiration as well as decreasing chest wall compliance [2]. Obesity is also associated with increased levels of circulating pro-inflammatory markers such as CRP, IL6, TNFalpha and other cytokines [36]. Systemic inflammation may explain some of the associations of obesity with impaired lung function. We indeed found that a small proportion (2%) of the effect of BMI on FVC was mediated by CRP, and 8.8% of the BMI effect on FEV1 was explained by inflammatory mechanisms (Figs. 1B and 4) suggesting indirect effects of BMI on airway obstruction through systemic inflammation.

### Type 2 diabetes

Cross-sectional and longitudinal studies have shown that middle-aged adults with T2D have worse lung function and slightly increased lung function decline [37]. The proposed mechanisms are glycosylation of collagen within the lung, decreased muscle strength, impacts on surfactant proteins and low grade inflammation [5]. Our MR supports the epidemiological observations confirming that T2D causally affects lung function (Fig. 3A), independent of associations with BMI. We also observed low-grade inflammation playing a key role in T2D lung function relationship as 14.9% of the T2D effect on FVC can be attributed to inflammatory mechanisms (Fig. 4).

Similar to BMI, we observed the causal effect of T2D having restrictive effects on the lung rather than obstructive (Fig. 3). These associations persist when accounting for other cardio-metabolic traits. However, we do see an attenuation of the T2D effect on FVC when adding systolic blood pressure or pulse pressure to the MMR model.

Some longitudinal observational studies have reported that low lung function is an independent predictor of incident T2D [37, 38], and there is evidence that even in non-diabetics, higher fasting glucose is associated with lower lung function [39]. We found no evidence of causal associations of lung function on T2D, but the presence of low lung function in ‘prediabetics’ has been postulated to be related to lifestyle or environmental factors in utero, early childhood or adolescence that predispose individual to increased risks of diabetes and low lung function in the future [39]. Our analysis is unable to address this hypothesis further.

### Inflammatory mechanisms and blood lipid levels

Our proxy for systemic inflammation in this study is serum CRP. In this context, we acknowledge that altered serum CRP levels may be affected by factors not discussed in this study such as infection or immune disease. We observed statistically significant effects of CRP on FVC (Fig. 3). These associations were attenuated, but remained significant, after adjustment for BMI, pulse pressure or total cholesterol (Fig. 4). This implies that CRP may contribute to impaired lung function as shown by many epidemiological studies [3, 4, 40].

Observational studies of associations of lipid levels and the presence of COPD have been inconsistent and a recent meta-analysis found no evidence for associations of COPD with serum levels of HDL-C, LDL-C, TC and TG [41]. In cross-sectional studies, any association may be masked by lipid-lowering treatments—and there may be underlying associations of COPD with high triglyceride levels [41]. A large population-based cross-sectional study in France showed strong associations of restrictive lung function deficits with cardio-metabolic, reporting associations with lipid profile [2]. Our analysis does not support a causal effect of total cholesterol and triglycerides on lung function (Additional File 1: Fig. S8-S10).

### Coronary artery disease and blood pressure

Association between lung function and CAD, suggesting an effect of lung function on CAD, have not been stable across sensitivity analysis (Additional File 1: Fig. S12, S13); however, they are in line with a recent study by Marouli et al. [42] suggesting that lung function may be a mediator of the effect of standing height on CAD. These observations make an association of lung function with blood pressure more likely, however still difficult to interpret, mirroring the inconsistency that has been seen in large observational studies. Overall, our MR seems to suggest a causal effect of FEV1/FVC on blood pressure (Fig. 3B, Additional File 1: Fig. S10-S13). ‘High blood pressure’ (systolic greater than 130mmHg or diastolic greater than 85mm Hg) has been associated with lower FEV1 and FVC in NHANES III [43], systolic blood pressure has been associated with lower FVC in the 2001 Korean National Health and Nutrition Survey (KNHNS) [44] and with COPD (defined by smoking status and airway obstruction) in the later fifth KNHNS V [45]. One report suggests associations may be explained by the use of antihypertensive medication [46]. Our analysis supports a genetic correlation between pulse pressure and FEV1, but none with diastolic or systolic pressure; while MR analysis shows a causal effect of FEV1/FVC on blood pressure (Fig. 3) [46]. More studies are needed to confirm and understand these associations.

### Impact on public health

We hope that the presented findings will increase awareness of the relationship between lung function and cardiometabolic disease amongst clinicians, particularly general practitioners, and encourage clinicians to regularly conduct spirometry on their patients even amongst those without symptoms of lung disease. Furthermore, this work highlights the notion that efforts to reduce obesity and T2D will also improve lung function and lung health. Thus, measures taken to reduce obesity in the general population can also be viewed as measures to improve lung health. Finally, this study also suggests weight loss as a measure to improve respiratory health.

### Mendelian randomisation

Mendelian randomisation analysis are a great tool to use large-scale GWAS results to gain public health relevant insights; however, one of the major limitations in MR is weak instrument bias, meaning the variant explains little variation of the exposure. To overcome this source of bias, we selected variants from large-scale GWAS (Table 1) in combination with an a priori defined threshold of 5×10^-8^ (Additional File 2: Table S3). Additionally, we performed a power analysis, which showed that this MR analysis was sufficiently powered (Additional File 1: Fig. S15).

Another major challenge in this MR study is pleiotropy, the potential for a SNP used as instrument for a risk factor to affect more than one phenotype. Due to this complexity in the present MR study, we relied on consistency of results of multiple MR methods with different assumptions as well as results from multivariable MR. [18] We used very common IVW MR method to create a precise reference estimate which, however, is vulnerable to pleiotropy and extreme values. As second more robust method (Fig. 3), we used weighted median-based method, which is robust to outliers and works even if up to 50% of variants are invalid. Third method in use was mode-based estimation (MBE) which is another consensus-based method (Additional File 1: Supplementary methods) with similar properties as weighted median method. MBE relies on the so-called Zero Modal Pleiotropy Assumption [47]. That means that even if the group of valid instruments is only 40%, those will make the largest group of estimates within the distribution of ratio estimates and thus be driving the causal estimate. Our fourth estimate (Fig. 3) originates from robust MR Egger method (Fig. 3) and is very common in MR literature [14, 48, 49]. It attempts to model pleiotropy under the InSIDE (instrument strength independent of direct effect) [50], which assumes that pleiotropic effects need to be uncorrelated with each other—an assumption that may not be met by all traits in our analysis. Additionally, we performed sensitivity analysis applying outlier robust approaches and excluding potentially pleiotropic and/or invalid instruments from the analysis. These recently developed methods MR-PRESSO [27] and CONMIX [28] attempt to model pleiotropy in MR analysis and provide a measure of pleiotropy for each SNP. We excluded SNPs flagged by these methods re-analysed the data and observed generally lower *P* values for the associations presented in the study (Additional File 1: Fig. S16-S21, Additional File 2: Table S3).

## Conclusions

In conclusion, we provide evidence for genetic correlations between BMI, CRP, T2D and coronary artery disease with FEV_1_, FVC and their ratio. These correlations reflect causal associations for the effects of BMI on all lung parameters and for T2D on FVC and FEV1/FVC. These associations are broadly independent from each other and of other metabolic traits with a small proportion of the effect of T2D and BMI on impaired lung function being mediated by serum CRP. There was evidence that FEV1/FVC ratio have a causal effect on blood pressure but not on the other tested cardio-metabolic traits. Our results strongly support efforts to reduce obesity and T2D as measures to improve lung function and lung health in the general population.

## Supplementary Information

**Additional file 1. **This file contains supplementary methods describing the analysis in more detail. **Table S1.** Summary of analyzed NFBC1966 data. **Table S2.** published datasets used in this study. **Table S5.** Cohort characteristic of UK Biobank. **Table S8.** Regression results between lung function and cardiometabolic traits in NFBC1966. **Table S9.** Results of LD-score regression. **Figure S3.** Correlation and Principal component analysis in NFBC1966. **Figure S4.** Forest plot of multivariable MR with anthropomorphic trats and smoking added to the model. **Figure S8.** Forest plots showing effect of cardio-metabolic traits on FEV1. **Figure S9.** Forest plots showing effect of cardio-metabolic traits on FVC. **Figure S10.** Forest plots showing effect of cardio-metabolic traits on FEV1pFVC. **Figure S11.** Forest plots showing effect of FEV1 on cardio-metabolic traits. **Figure S12.** Forest plots showing effect of FVC on cardio-metabolic traits. **Figure S13.** Forest plots showing effect of FEV1pFVC on cardio-metabolic traits. **Figure S14.** Forest plots of sex stratified analysis. **Figure S15.** Power analysis. **Figure S16**. Sensitivity analysis: Forest plots showing effect of cardio-metabolic traits on FEV1. **Figure S17.** Sensitivity analysis: Forest plots showing effect of cardio-metabolic traits on FVC. **Figure S18.** Sensitivity analysis: Forest plots showing effect of cardio-metabolic traits on FEV1pFVC. **Figure S19.** Sensitivity analysis: Forest plots showing effect of FEV1 on cardio-metabolic traits. **Figure S20.** Sensitivity analysis: Forest plots showing effect of FVC on cardio-metabolic traits. **Figure S21.** Sensitivity analysis: Forest plots showing effect of FEV1pFVC on cardio-metabolic traits.

**Additional file2: Table S3.** contains a detailed overview of all variants used as instruments in this study including chromosomal position, nearest gene, allele frequencies and *P* value of association to all tested cardiometabolic traits. **Table S4.** Linkage disequilibrium between SNPs subdivided into traits and chromosomes. **Table S6.** Sensitivity analysis including effect estimates, standard errors and *P*-values for associations between all exposures and all outcomes for all applied MR techniques. **Table S7.** Multivariable MR including effect estimates, standard errors from Multivariable MR. **Table S10.** Effect attenuation gives differences in effect size estimates when adding an additional predictor to multivariable MR analysis. **Table S11.** Sex stratified analysis including effect estimates, standard errors and *P*-values.

## Data Availability

The following summary statistics analysed in this study are publicly available: BMI Locke et al. [8], 2015, height, waist to hip ratio Randall et al. [51], 2013: (http://portals.broadinstitute.org/collaboration/giant/index.php/GIANT_consortium_data_files); T2D Scott et al. [9], 2017: (http://diagram-consortium.org/downloads.html ); Blood lipid levels, Willer et al. [11], 2013: (http://lipidgenetics.org ); Blood pressure Wain et al. [12], 2017: (https://www.ncbi.nlm.nih.gov/projects/gap/cgi-bin/study.cgi?study_id=phs000585.v1.p1); CAD Nikpay et al. [13], 2015: (http://www.cardiogramplusc4d.org/data-downloads/ ). Drinks per week, cigarettes per day Liu et al. [20], 2019: (https://conservancy.umn.edu/handle/11299/201564 ) The following analysed in this study are available upon request: CRP Dehghan et al. [10], 2011: Summary statistics are available from the corresponding author on request. UK Biobank data (Application Number 19136) are available upon request (https://www.ukbiobank.ac.uk/register-apply/) NFBC data is available from the University of Oulu, Infrastructure for Population Studies. Permission to use the data can be applied for research purposes via electronic material request portal. In the use of data, we follow the EU general data protection regulation (679/2016) and Finnish Data Protection Act. The use of personal data is based on cohort participant’s written informed consent at his/her latest follow-up study, which may cause limitations to its use. Please, contact NFBC project centre (NFBCprojectcenter@oulu.fi) and visit the cohort website (www.oulu.fi/nfbc) for more information.

## Abbreviations

BMI: Body mass index
CAD: Coronary artery disease
CRP: C-reactive protein
DBP: Diastolic blood pressure
FEV1: Forced expiratory volume in second one
FVC: Forced vital capacity
FEV1/FVC: Ratio of forced expiratory volume in second one and forced vital capacity
HDL-C: High-density lipoprotein cholesterol
LDL-C: Low-density lipoprotein cholesterol
LD-score regression: Linkage disequilibrium score regression
MR: Mendelian randomisation
MMR: Multivariable Mendelian randomisation
PP: Pulse pressure
SBP: Systolic blood pressure
SNP: Single nucleotide polymorphism
TC: Total cholesterol
TG: Triglycerides
T2D: Type 2 diabetes
