# Supplementary material for: Genetic correlation and causal relationships between cardio-metabolic traits and lung function impairment
Source: Genome Med. 2021 Jun 21;13:104. doi: 10.1186/s13073-021-00914-x (PMC8215837; doi:10.1186/s13073-021-00914-x)
Supplement: Supplementary file 1 — Additional file 1. This file contains supplementary methods describing the analysis in more detail. Table S1. Summary of analyzed NFBC1966 data. Table S2. published datasets used in this study. Table S5. Cohort characteristic of UK Biobank. Table S8. Regression results between lung function and cardiometabolic traits in NFBC1966. Table S9. Results of LD-score regression. Figure S3. Correlation and Principal component analysis in NFBC1966. Figure S4. Forest plot of multivariable MR with anthropomorphic trats and smoking added to the model. Figure S8. Forest plots showing effect of cardio-metabolic traits on FEV1. Figure S9. Forest plots showing effect of cardio-metabolic traits on FVC. Figure S10. Forest plots showing effect of cardio-metabolic traits on FEV1pFVC. Figure S11. Forest plots showing effect of FEV1 on cardio-metabolic traits. Figure S12. Forest plots showing effect of FVC on cardio-metabolic traits. Figure S13. Forest plots showing effect of FEV1pFVC on cardio-metabolic traits. Figure S14. Forest plots of sex stratified analysis. Figure S15. Power analysis. Figure S16. Sensitivity analysis: Forest plots showing effect of cardio-metabolic traits on FEV1. Figure S17. Sensitivity analysis: Forest plots showing effect of cardio-metabolic traits on FVC. Figure S18. Sensitivity analysis: Forest plots showing effect of cardio-metabolic traits on FEV1pFVC. Figure S19. Sensitivity analysis: Forest plots showing effect of FEV1 on cardio-metabolic traits. Figure S20. Sensitivity analysis: Forest plots showing effect of FVC on cardio-metabolic traits. Figure S21. Sensitivity analysis: Forest plots showing effect of FEV1pFVC on cardio-metabolic traits. [file 13073_2021_914_MOESM1_ESM.docx]

Additional File 1

Genetic correlation and causal relationships between cardio-metabolic traits and Lung function Impairment

Matthias Wielscher^1^, Andre F.S. Amaral^2^, Diana van der Plaat^2^, Louise V. Wain^3,4^, Sylvain Sebert^5,6^, David Mosen-Ansorena^1^, Juha Auvinen^5,6^, Karl-Heinz Herzig^6,7,8^, Abbas Dehghan^1^, Debbie L Jarvis^2#^, Marjo-Riitta Jarvelin^1,5,6,9#^

1 Department of Epidemiology and Biostatistics, MRC-PHE Centre for Environment and Health, School of Public Health, Imperial College London, Norfolk Place, London, W2 1PG, UK

2 National heart and lung institute (NHLI), Imperial College London, Emmanuel Kaye Building, London, SW3 6LR, UK.

3 Genetic Epidemiology Group, Department of Health Sciences, George Davies Centre, University of Leicester, University Rd, Leicester LE1 7RH, UK

4 National Institute for Health Research, Leicester Respiratory Biomedical Research Centre, Glenfield Hospital, Leicester, University Rd, Leicester LE1 7RH, UK

5 Center for Life Course Health Research, Faculty of Medicine, University of Oulu, P.O.Box 8000, FI-90014 Oulu, Finland

6 Biocenter of Oulu, University of Oulu, Aapistie 5, FI-90014 Oulu, Finland.

7 Research Unit of Biomedicine, Medical Research Center (MRC), University of Oulu, University Hospital, Oulu, P.O. Box 8000, Finland

8 Department of Gastroenterology and Metabolism, Poznan University of Medical Sciences, 41 Jackowskiego St., 60-512 Poznan, Poland

9 Department of Life Sciences, College of Health and Life Sciences, Brunel University London, Kingston Lane, London UB8 3PH, UK

# corresponding authors: [m.jarvelin@imperial.ac.uk](mailto:m.jarvelin@imperial.ac.uk) and [d.jarvis@imperial.ac.uk](mailto:d.jarvis@imperial.ac.uk)

**Additional file 1: Supplementary methods:**

**SNP -** **cardio-metabolic traits associations.**

We extracted the effect estimates for SNPs associated (P< 5x10-8) with BMI, T2D, CRP, blood lipids, blood pressure and CAD from publicly available lists. These summary statistics resulted from large meta-analyses of at least 80,000 individuals (Table 1). The estimates for BMI-SNPs associations are from a GIANT consortium study ([1](#_ENREF_1)), blood pressure associated SNPs are from a study by Wain et al. ([2](#_ENREF_2)), blood lipid associated SNPs are from the Global Lipids Genetics consortium ([3](#_ENREF_3)), CAD associated SNPs are from the Cardiogram consortium ([4](#_ENREF_4)), T2D associated SNPs are from the DIAGRAM consortium ([5](#_ENREF_5)), and CRP associated SNPs are from a study by Dehghan et al. ([6](#_ENREF_6)). Individual summary statistics underwent study specific QC as well as genomic control procedure. Genomic coordinates were lifted over to GRCh37/hg19 if necessary.

**SNP-lung function associations**.

We used the UK Biobank release from July 2017. We restricted to HRC imputed SNPs due to possible wrong genomic position of non-HRC imputed SNPs. Genotyped SNPs were filtered for MAF > 5%, HWE P > 1x10-6, and missingness < 0.015, to estimate the kinship matrix. Genetic association results were retrieved for ~7.1 million SNPs remaining after filtering the HRC imputed data. The association tests were performed using linear mixed models (LMM) for all typed and imputed SNPs in dosage format using the BOLT-LMM (v2.3) software ([7](#_ENREF_7)), which corrects for population structure and cryptic relatedness. We assumed an additive mode of inheritance.

Lung function measurements in the UK Biobank were generated with a Vitalograph Pneumotrac 6800 spirometer (Vitalograph Ltd., Maids Moreton, UK) according to recommendations. The present analysis is based on the best (highest) available lung function measure from those who performed at least two acceptable blows and where both FEV1 and FVC, although not from the same blow, were reproducible within 150 mL, as recommended by the American Thoracic Society/European Respiratory Society (ATS/ERS)([8](#_ENREF_8)).

**Cross trait LD score regression**

All publicly available summary statistics (Table 1) were corrected for genomic inflation. We used the LD score regression y-intercept as correction factor for genomic control procedure of the genome-wide association results obtained from the UK Biobank([9](#_ENREF_9)). We estimated pairwise genetic correlation and heritability using the recommended settings in LD score software (v1.0.0) ([10](#_ENREF_10)).

**Mendelian Randomization**

Mendelian Randomization relies on Mendel’s second law, that is, genotypes are assorted at random. This balances confounding and excludes reverse causation. A polymorphism associated with both the risk factor and with impaired lung function supports a causal role of the risk factor.([11](#_ENREF_11)). We performed a 2-sample Mendelian Randomization. If not indicated otherwise, MR analyses were performed using the CRAN package *Mendelianrandomization* ([12](#_ENREF_12)).

***IVW MR:*** We estimated the causal effect of the risk factor on lung function using widely used IVW MR. We combined inverse variance weighted ratios of genetic variants using a fixed effect meta-analysis model ([13](#_ENREF_13)). We retrieved IVW Estimates and P values as well Cochran‘s Q and a P value for heterogeneity as indication of Pleiotropy (Additional File 2: Table S6). Briefly, if all genes represent valid instruments, their MR estimates should vary only by chance. The presence of pleiotropy is then investigated by using the between‐instrument heterogeneity Q test derived separately for each instrument([14](#_ENREF_14)). The P value tests the null hypothesis that all genetic variants are estimating the same causal parameter; rejection of the null is an indication that one or more variants may be pleiotropic. For IVW BMI effect size estimates, we performed linear regression analyses between the SNPs and BMI (N=453,868) adjusted for age, age^2^, centre, first 10 principal components (PCs) and genotyping batch (UK BiLEVE array and the UK Biobank Axiom array (2 data releases)).

**Weighted Median method:** Similar to IVW method, however using the median of the ratio estimates as opposed to the mean in IVW. In the weighted version we add a weight corresponding to the precision of the ratios. The method will provide unbiased estimates even if up to 50% of the variants are invalid instruments.

**Mode based estimation (MBE):** The causal estimate is the maximum of a density function that is constructed from a normal density of each genetic variant. Similar to weighted median method this method is robust to outliers and has a higher break point as IVW and MR Egger method. Mode based estimation method is also less sensitive to violation of the Instrument Strength Independent of Direct Effect (InSIDE) assumptions([15](#_ENREF_15)), because it relaxes the instrumental variable assumptions.

**MR Egger:** MR Egger method attempts to model the distribution of invalid instruments. We regress the effect sizes of the variant-outcome associations against effect sizes of variant-exposure associations. An unconstrained interception term should reflect pleiotropy and removes the assumption that all instrumental variables are valid.

**Sensitivity analysis:** We performed penalized versions for some of the methods mentioned above. Furthermore, we rerun the analysis with an altered set input SNPs based on outlier SNPs detected by either MR-PRESSO([16](#_ENREF_16)) or the contamination mixture method([17](#_ENREF_17)). MR PRESSO was developed to detect horizontal pleiotropy. The variants effects on the outcome are regressed against the variant effects on the exposure. Again, regression line is the causal estimate. Then the same regression is performed without the tested variant and a residual sum of squares is calculated as the difference between regression lines. To obtain a P-value specific for pleiotropy of every variant a H0 distribution of RSS is simulated assuming no horizontal pleiotropy. Then we rerun IVW MR method without the variants flagged up by MR-PRESSO. Similar we run contamination mixture method to create a subset of SNPs to use in our sensitivity analysis. Contamination mixture method constructs a likelihood function based on the variant specific causal estimates if the instrument is valid the causal estimate of the tested variant will be around this estimate if not it will be around 0 with a high standard deviation. Based on the contribution of each variant to the likelihood the tested variant will be rated valid of invalid.

**Stratified Analysis:**

To investigate whether or not effects of tested risk factors are sex specific, we performed a stratified analysis. We performed lung function association tests in UKBioBank separately for male and female participants, generating 6 sets of summary statistics. We then performed our standard MR analysis of BMI, CRP T2D with the result of the sex stratified lung function GWAS (Additional File1: Figure S14, Additional File 2: S11).

**Multivariable MR:** To account for the high genetic correlation between the different cardio-metabolic traits (Additional File 1: Table S9), we used a recently developed extension of MR, called multivariable MR([18](#_ENREF_18)). We regressed the coefficients for the SNP-outcome association against all risk factors simultaneously and used the residuals of that regression as outcome to estimate the causal effect. We used the weighted regression-based approach to achieve this([19](#_ENREF_19)). Too examine whether our findings were influenced by alcohol or tobacco addiction, height as well as waist to hip related pleiotropic effects, we retrieved summary statistics from <https://conservancy.umn.edu/handle/11299/20156> for addiction traits and <https://portals.broadinstitute.org/collaboration/giant/index.php/GIANT_consortium_data_files> for anthropomorphic traits and included them in multivariable MR analysis. (Additional File 1 Figure S14)

***Bidirectional MR:*** We repeated the MR in the opposite direction to determine possible causal effects of lung function on cardio-metabolic traits (Figure 1). For this we used a set of validated SNPs described by Wain et al ([20](#_ENREF_20)) as instruments for lung function and performed a multivariable MR and IVW MR (Additional File 2, Table S6, S7).

**Instrument strength and Power analysis.** We calculated the cumulative variance explained applying the formula VarExp = beta^2 (1 – f) 2f, where beta is the coefficient for the SNP association to risk factor and f is the effect allele frequency. For this calculation as well as for Mendelian Randomization we included the most significant variant within a 1MB window out of a pool of variants with a P-value less than 5E-08. On a genome-wide level we calculated the genomic inflation factor lambda and extracted LD score intercept as indicator of the association between the risk or outcome and the genotype. Power calculations for our MR analysis were done specifically for binary and continuous outcomes ([21](#_ENREF_21), [22](#_ENREF_22)) The parameters for these calculations was the actual sample numbers of the outcome summary statistic, the cumulative variance explained as effect size of the risk factor and for the alpha level we used 2xE-03, the Bonferroni adjusted significance threshold used in this study. Power was calculated for varying true causal effect estimates ranging from 0.01 to 0.2.

**Additional File 1 tables:**

**Additional File 1 Table S1** NFBC1966 cohort characteristics (N = 5567) for clinical examination at participants age 46. Values given as percent of the total sample count or mean and standard error is given.

|  | N (perc) or mean (SD) |
| --- | --- |
| male | 2451 (44) |
| female | 3116 (55.9) |
| current smokers | 811 (15.4) |
| Former smokers | 2056 (38.9) |
| Never smokers | 2409 (45.6) |
| Pack-years | 10.4 (9.5) |
| Age (years) | 46.6 (0.6) |
| Height (cm) | 170.8 (9.1) |
| FEV1 (L) | 3.43 (0.71) |
| FVC (L) | 4.44 (0.96) |
| FEV1/FVC | 0.77 (0.06) |
| BMI | 26.8 (4.8) |
| CRP [mg/L] | 1.5 (2.8) |
| SBP | 128.8 (17.3) |
| DBP | 86 (11.2) |
| PP | 42.7 (10.4) |
| TC [mmol/L] | 5.3 (0.9) |
| TG [mmol/L] | 1.2 (0.8) |
| LDL [mmol/L] | 3.4 (0.6) |
| HDL [mmol/L] | 1.5 (0.3) |
| CAD cases | 136 (2.5) |
| CAD controls | 5128 (97.4) |
| T2D cases | 151 (3.1) |
| T2D controls | 4814 (96.9) |

**Additional File 1 Table S2:** Characteristics of summary statistics and Instrument strength; *validated loci used for inverse direction (described by Wain et al.([20](#_ENREF_20))); **reported in study; ***European ancestry. Source gives the publication describing the discovery of the instruments. Lambda GC values stands for lung function values are before genomic control correction. LDSC intercept is the y interception from Linkage disequilibrium score regression, which can be interpreted as indicator of genomic inflation. N SNPs is the number of SNPs reported as genome wide significant in published GWAS study. Cumulative variance is the variance explained by genetic variants discovered in each study (i.e. instruments for Mendelian Randomisation)

| **trait** | **source** | **lambda GC** | **LDSC intercept** | **N SNPs< 5xE-08** | **cumulative variance** |
| --- | --- | --- | --- | --- | --- |
| FEV1 | UKBB & Wain et al. | 1.55 | 1.1 | 232 (32*) | 0.096** |
| FVC | UKBB & Wain et al. | 1.6 | 1.12 | 227 (21*) | 0.064** |
| FEV1/FVC | UKBB & Wain et al. | 1.55 | 1.1 | 332 (59*) | 0.143** |
| BMI | Locke et al. | 1.08 | 0.88 | 77*** | 0.027** |
| DBP | Wain et al. | 0.99 | 0.85 | 54 | 0.013 |
| SBP | Wain et al. | 1 | 0.87 | 55 | 0.013 |
| PP | Wain et al. | 1 | 0.87 | 31 | 0.005 |
| CAD | Nikpay et al. | 1.03 | 0.9 | 48 | 0.133** |
| CRP | Dehghan et al. | 1.09 | 1 | 18 | 0.05** |
| HDL | Willer et al. | 1.01 | 0.91 | 106 | 0.017 |
| LDL | Willer et al. | 1.01 | 0.98 | 82 | 0.023 |
| TG | Willer et al. | 1.01 | 0.98 | 66 | 0.013 |
| TC | Willer et al. | 1 | 0.96 | 102 | 0.023 |
| T2D | Scott et al. | 1.07 | 1.01 | 28 | 0.045 |

**Additional File 1 *Table S5*:** UK Biobank cohort characteristics (N = 270,381). Values given as percent of the total sample count or mean and standard error is given. To give an overview of medications that may influence cardio-metabolic traits , we analysed UKBB variable “Medication for cholesterol, blood pressure, diabetes, or take exogenous hormones”, for overview on incident T2D, and heart disease in the studied patient cohort we analysed the variable“ Non-cancer illness code, self-reported”

|  | **% or mean (SD)** |
| --- | --- |
| male | 42.8 % |
| female | 57.2 % |
| current smokers | 10.1 % |
| Former smokers | 36.8 % |
| Never smokers | 53.1 % |
| Pack-years | 22.8 (18.1) |
| Age (years) | 56.5 (8.0) |
| Height (cm) | 1.68 (0.09) |
| FEV1 (L) | 2.81 (0.75) |
| FVC (L) | 3.71 (0.94) |
| FEV1/FVC | 0.76 (0.06) |
| shortness of breath walking on level ground | 9.39 % |
| BMI | 27.3 (4.71) |
| Obese (BMI > 30) | 23.4 % |
| SBP (mmHg) | 139.8 (19.6) |
| DBP (mmHg) | 82.1 (10.6) |
| Hypertension | 29.3 % |
| Cholesterol lowering medication | 11.9 % |
| Blood pressure medication | 9.7% |
| Insulin | 0.12% |
| type 2 diabetes | 0.19% |
| type 1 diabetes | 0.05% |
| diabetes | 1.28% |
| heart/cardiac problem | 0.11% |
| heart attack/myocardial infarction | 2.51% |


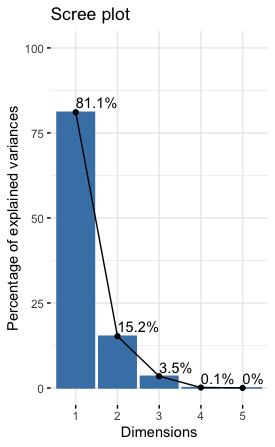

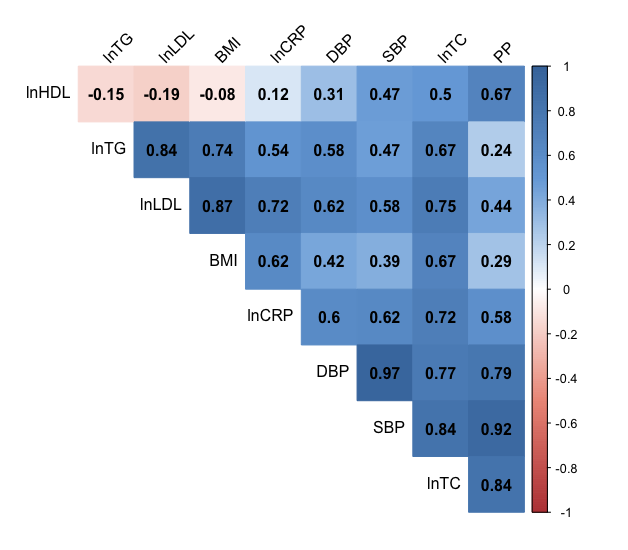


**Additional File 1 Figure S3: Correlation and Principal component analysis in NFBC1966.** Correlation structure of cardiometabolic traits in NFBC1966. Dichotomous traits CAD and T2D were excluded from correlation analysis. Principal Component Analysis of 11 cardio- metabolic traits in NFBC1966. Plot show variance explained by each Principal component. More than 99% of the variance is explained by first 3 principal components.


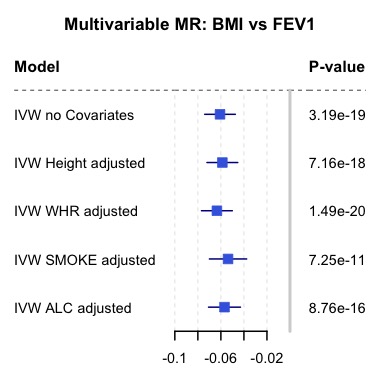

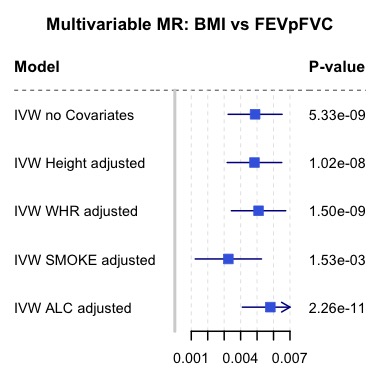

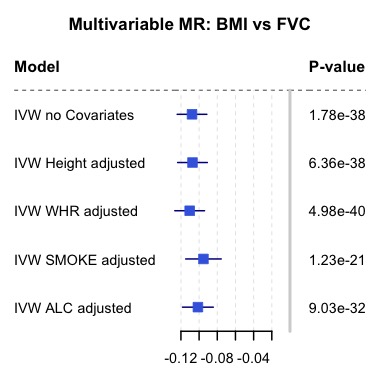

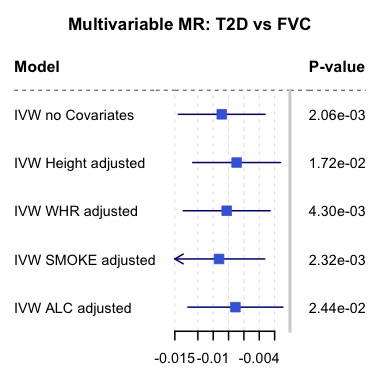

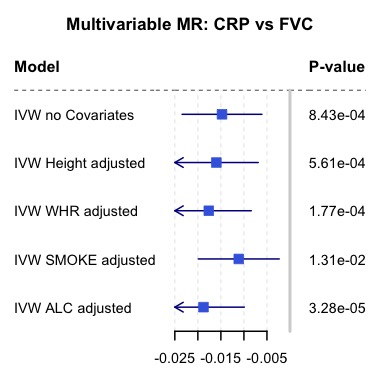

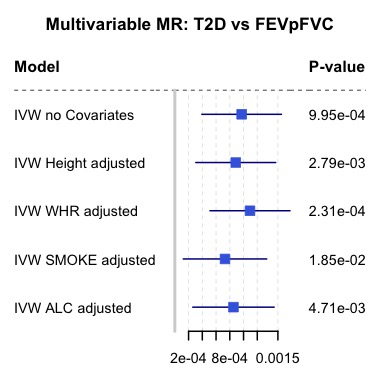


**Additional File 1 Figure S4 Additional pleiotropy inspection.** We retrieved data for height from GIANT homepage and data for alcohol and smoking here (). Data were harmonized with our existing SNP lists and multivariable Mendelian Randomization was repeated for selected risk factors. Forest plots show the influence of these traits on the instruments for BMI, CRP and T2D.

**Additional File 1 Table S8:** Association between cardio-metabolic traits and lung function recorded in observational data retrieved from NFBC1966. Clinical assessment at participants age 46.

| Risk factor | Outcome | Estimate | SE | P-value | N |
| --- | --- | --- | --- | --- | --- |
| BMI | **FVC** | -0.019 | 0.002 | 9.73E-37 | 5293 |
| DBP | **FVC** | -0.004 | 0.001 | 1.18E-10 | 5287 |
| SBP | **FVC** | -0.002 | 0.000 | 4.93E-07 | 5287 |
| PP | **FVC** | -0.001 | 0.001 | 2.27E-01 | 5287 |
| CAD | **FVC** | -0.063 | 0.047 | 1.79E-01 | 5258 |
| CRP | **FVC** | -0.109 | 0.008 | 1.27E-45 | 4984 |
| HDL | **FVC** | 0.278 | 0.032 | 5.44E-18 | 5277 |
| LDL | **FVC** | -0.048 | 0.027 | 7.72E-02 | 5277 |
| TG | **FVC** | -0.174 | 0.016 | 2.99E-28 | 5277 |
| TC | **FVC** | -0.012 | 0.043 | 7.77E-01 | 5275 |
| T2D | **FVC** | -0.302 | 0.031 | 5.85E-22 | 5259 |
| BMI | **FEV1** | -0.009 | 0.001 | 2.75E-13 | 5304 |
| DBP | **FEV1** | -0.002 | 0.001 | 3.43E-03 | 5299 |
| SBP | **FEV1** | -0.001 | 0.000 | 1.05E-02 | 5299 |
| PP | **FEV1** | -0.001 | 0.001 | 3.15E-01 | 5299 |
| CAD | **FEV1** | -0.034 | 0.038 | 3.67E-01 | 5270 |
| CRP | **FEV1** | -0.069 | 0.006 | 4.16E-28 | 4993 |
| HDL | **FEV1** | 0.140 | 0.026 | 1.16E-07 | 5286 |
| LDL | **FEV1** | -0.007 | 0.022 | 7.62E-01 | 5286 |
| TG | **FEV1** | -0.117 | 0.013 | 2.53E-19 | 5286 |
| TC | **FEV1** | 0.004 | 0.035 | 9.17E-01 | 5284 |
| T2D | **FEV1** | -0.184 | 0.026 | 1.02E-12 | 5271 |
| BMI | **FEV1/FVC** | 0.001 | 0.000 | 4.27E-13 | 5276 |
| DBP | **FEV1/FVC** | 0.0001 | 0.000 | 2.48E-06 | 5270 |
| SBP | **FEV1/FVC** | 0.0001 | 0.000 | 1.38E-03 | 5270 |
| PP | **FEV1/FVC** | 0.0001 | 0.000 | 9.31E-01 | 5270 |
| CAD | **FEV1/FVC** | 0.001 | 0.005 | 7.94E-01 | 5241 |
| CRP | **FEV1/FVC** | 0.004 | 0.001 | 1.46E-05 | 4968 |
| HDL | **FEV1/FVC** | -0.018 | 0.004 | 1.64E-06 | 5260 |
| LDL | **FEV1/FVC** | 0.008 | 0.003 | 1.19E-02 | 5260 |
| TG | **FEV1/FVC** | 0.004 | 0.002 | 2.27E-02 | 5260 |
| TC | **FEV1/FVC** | 0.003 | 0.005 | 5.26E-01 | 5258 |
| T2D | **FEV1/FVC** | 0.011 | 0.004 | 1.98E-03 | 5242 |

**Additional File 1 Table S9:** Result of cross trait LD score regression. Correlation values range from -1 to +1. Multiple testing threshold for this analysis is P < 0.0027.

| trait 1 | trait 2 | correlation | SE | Z score | P value |
| --- | --- | --- | --- | --- | --- |
| FEV1/FVC | BMI | 0.1512 | 0.0205 | 7.3597 | 1.84E-13 |
| FEV1/FVC | CAD | 0.0706 | 0.0267 | 2.642 | 0.0082 |
| FEV1/FVC | CRP | 0.0858 | 0.0443 | 1.9364 | 0.0528 |
| FEV1/FVC | DBP | -0.0248 | 0.0314 | -0.7893 | 0.4299 |
| FEV1/FVC | HDL | -0.1256 | 0.0442 | -2.8424 | 0.0045 |
| FEV1/FVC | LDL | 0.0409 | 0.033 | 1.2408 | 0.2147 |
| FEV1/FVC | PP | -0.0472 | 0.0301 | -1.569 | 0.1167 |
| FEV1/FVC | SBP | -0.0453 | 0.0303 | -1.4949 | 0.1349 |
| FEV1/FVC | T2D | 0.0146 | 0.0333 | 0.4375 | 0.6618 |
| FEV1/FVC | TC | 0.0319 | 0.0388 | 0.8226 | 0.4107 |
| FEV1/FVC | TG | 0.0943 | 0.0375 | 2.513 | 0.012 |
| FEV1 | BMI | -0.0694 | 0.0208 | -3.3362 | 0.0008 |
| FEV1 | CAD | -0.0725 | 0.0237 | -3.056 | 0.0022 |
| FEV1 | CRP | -0.1784 | 0.0388 | -4.5967 | 4.29E-06 |
| FEV1 | DBP | -0.0194 | 0.0307 | -0.6331 | 0.5267 |
| FEV1 | HDL | 0.0472 | 0.0457 | 1.0319 | 0.3021 |
| FEV1 | LDL | -0.0142 | 0.0307 | -0.4621 | 0.644 |
| FEV1 | PP | -0.0909 | 0.0291 | -3.121 | 0.0018 |
| FEV1 | SBP | -0.0726 | 0.0264 | -2.7551 | 0.0059 |
| FEV1 | T2D | -0.1947 | 0.0309 | -6.2925 | 3.12E-10 |
| FEV1 | TC | 0.008 | 0.033 | 0.243 | 0.808 |
| FEV1 | TG | -0.0114 | 0.0336 | -0.3394 | 0.7343 |
| FVC | BMI | -0.1643 | 0.0192 | -8.575 | 9.91E-18 |
| FVC | CAD | -0.1188 | 0.0255 | -4.6632 | 3.11E-06 |
| FVC | CRP | -0.2425 | 0.0357 | -6.7863 | 1.15E-11 |
| FVC | DBP | -0.0073 | 0.0291 | -0.2502 | 0.8024 |
| FVC | HDL | 0.131 | 0.0452 | 2.8977 | 0.0038 |
| FVC | LDL | -0.0463 | 0.0313 | -1.4791 | 0.1391 |
| FVC | PP | -0.0824 | 0.0285 | -2.892 | 0.0038 |
| FVC | SBP | -0.0591 | 0.0256 | -2.3099 | 0.0209 |
| FVC | T2D | -0.2376 | 0.0333 | -7.1422 | 9.19E-13 |
| FVC | TC | -0.0145 | 0.0312 | -0.4654 | 0.6417 |
| FVC | TG | -0.0694 | 0.0319 | -2.1764 | 0.0295 |


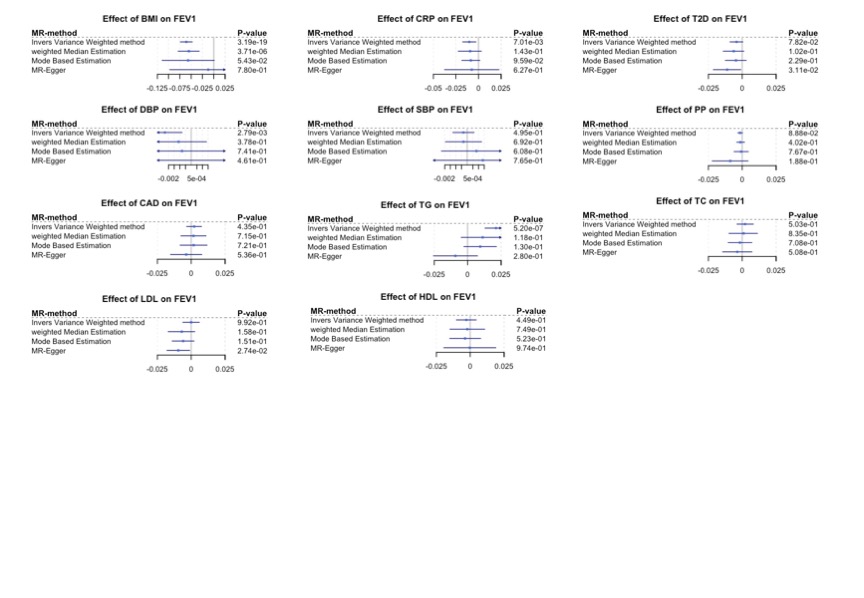


**Additional File 1 Figure S8 Forest plots showing effect of cardio-metabolic traits on FEV1.** Blue square represents causal estimate. Blue line is 95% confidence interval. Every line represents one approach to estimate the potential causal effect.


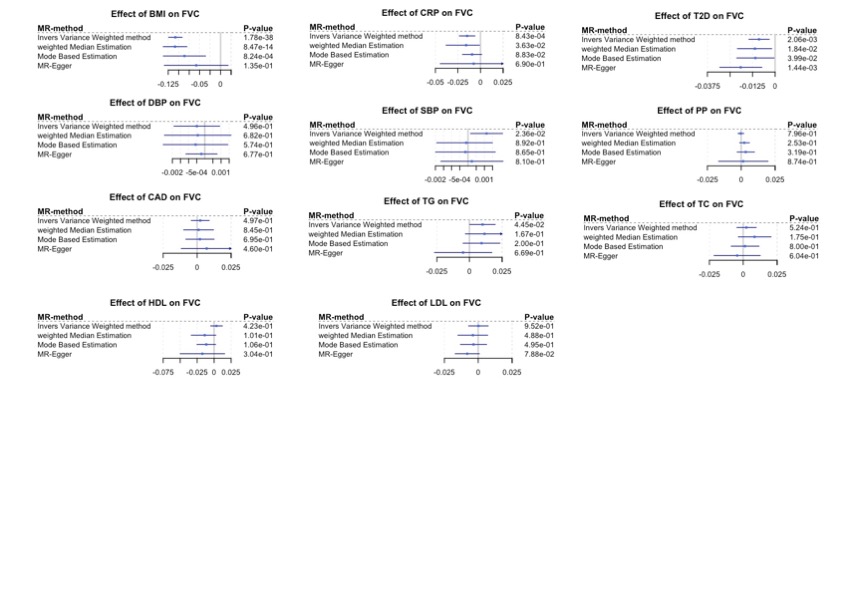


**Additional File 1 Figure S9: Forest plots showing effect of cardio-metabolic traits on FVC.** Blue square represents causal estimate. Blue line is 95% confidence interval. Every line represents one approach to estimate the potential causal effect.


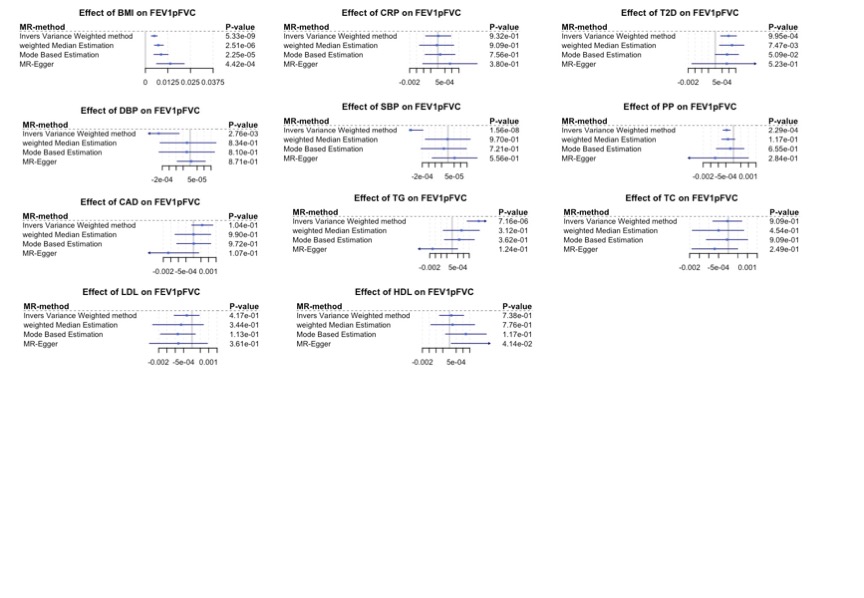


**Additional File 1 Figure S10: Forest plots showing effect of cardio-metabolic traits on FEV1pFVC.** Blue square represents causal estimate. Blue line is 95% confidence interval. Every line represents one approach to estimate the potential causal effect.


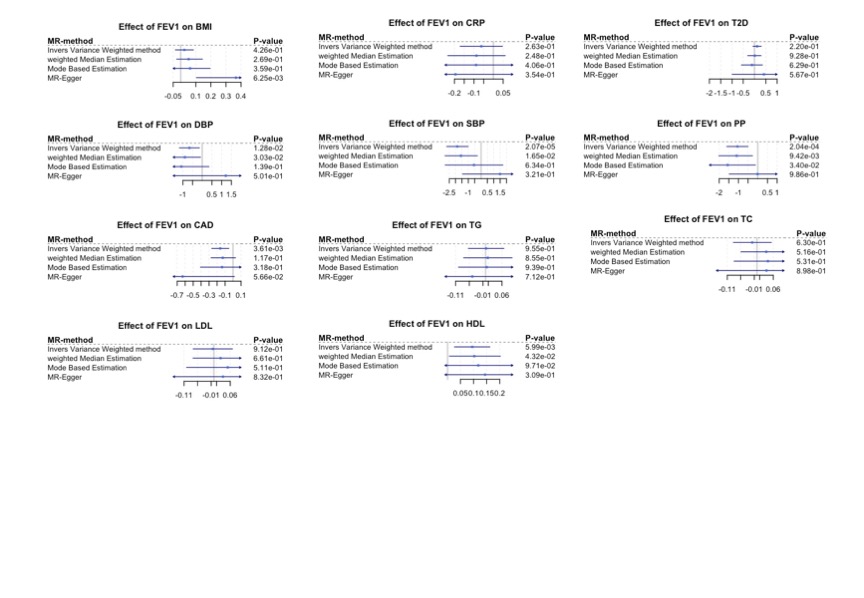


**Additional File 1 Figure S11: Forest plots showing effect of FEV1 on cardio-metabolic traits.** Blue square represents causal estimate. Blue line is 95% confidence interval. Every line represents one approach to estimate the potential causal effect.


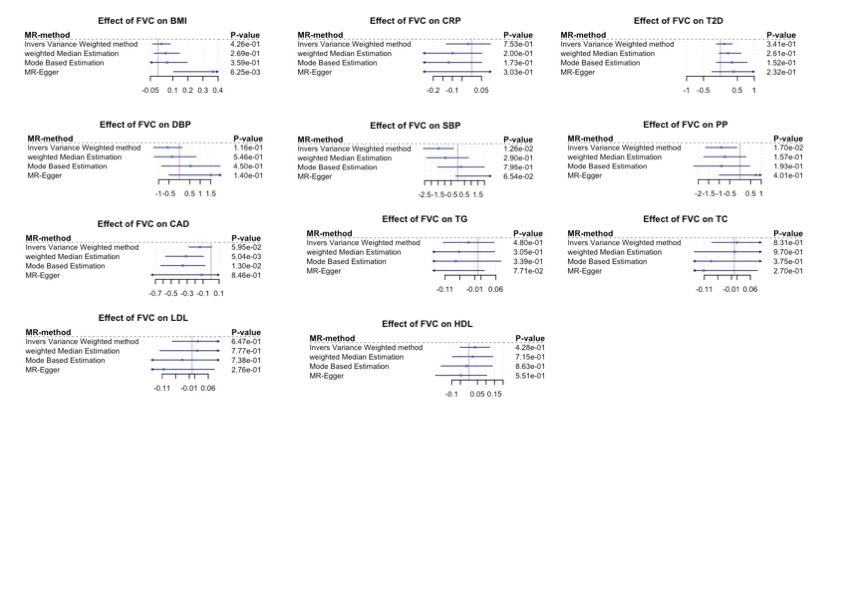


**Additional File 1 Figure S12: Forest plots showing effect of FVC on cardio-metabolic traits.** Blue square represents causal estimate. Blue line is 95% confidence interval. Every line represents one approach to estimate the potential causal effect.


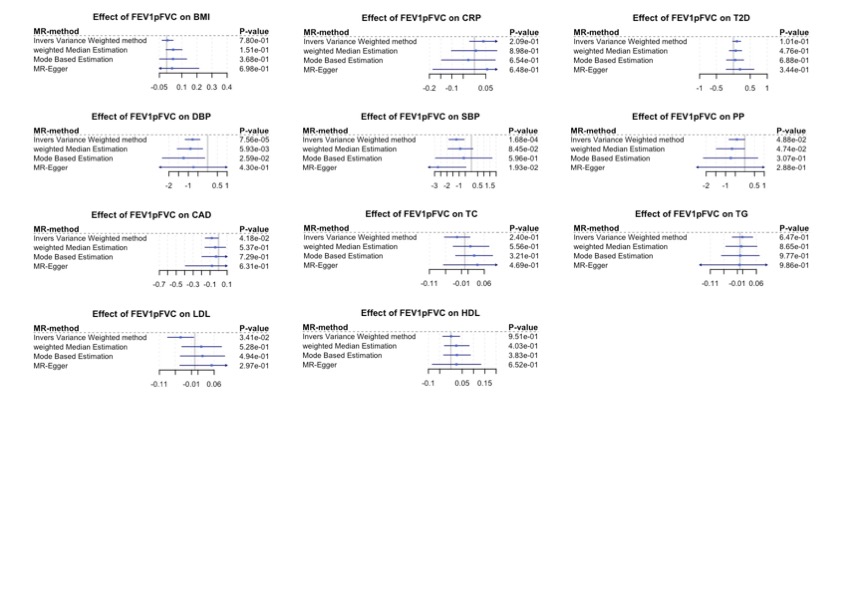


**Additional File 1 Figure S13: Forest plots showing effect of FEV1pFVC on cardio-metabolic traits.** Blue square represents causal estimate. Blue line is 95% confidence interval. Every line represents one approach to estimate the potential causal effect.


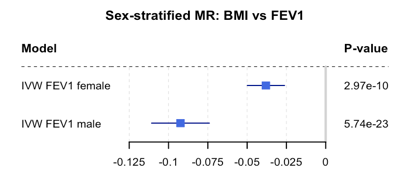

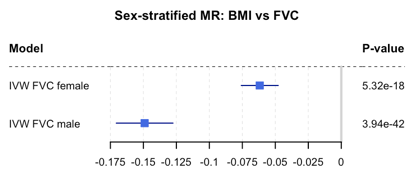

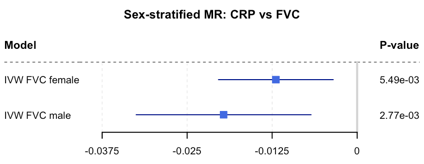

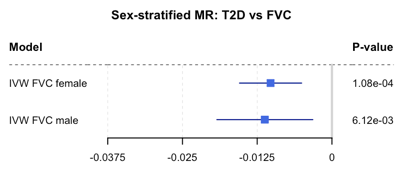

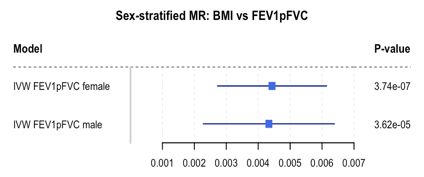

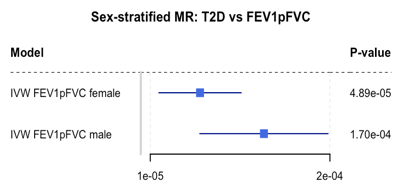


**Additional File 1 Figure S14: Sex stratified analysis.** We performed SNP lung function association tests in UKBioBank using BOLT LMM stratified for sex. This generated 6 sets of summary statistics, which were used for standard


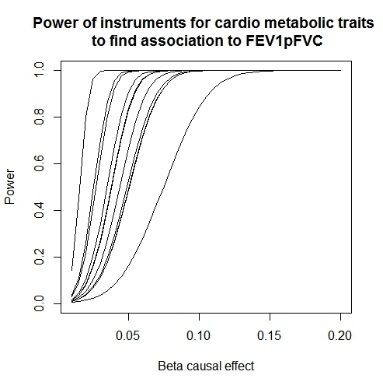

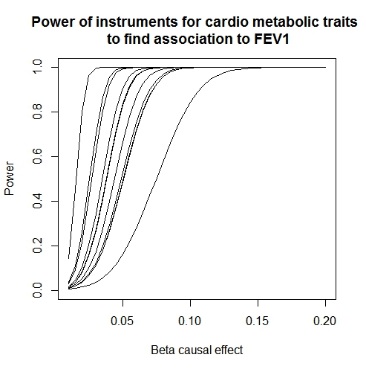

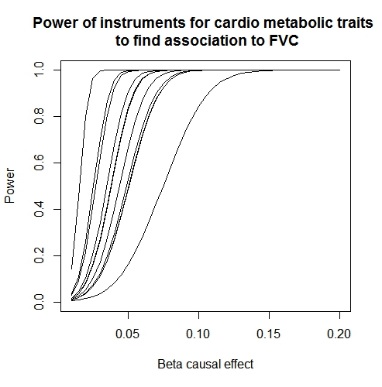

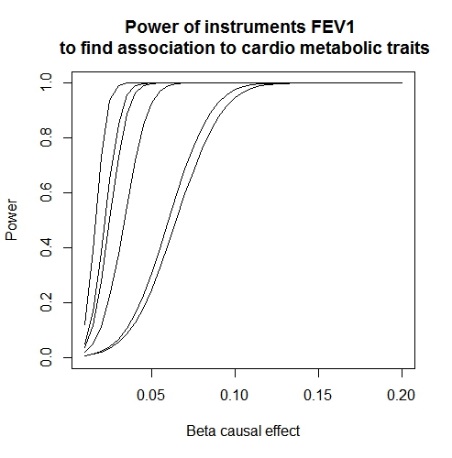


**Additional File 1 Figure S15:** Power analysis. Where possible we used the variance explained reported by the studies, otherwise we calculated the variance explained of the Poly Genic Risk score (PRS). We used an alpha level of 0.0028 and the actual sample numbers of the studies. For values at a causal effect estimate of 0.1.


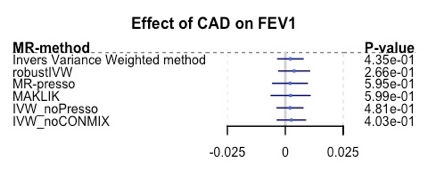

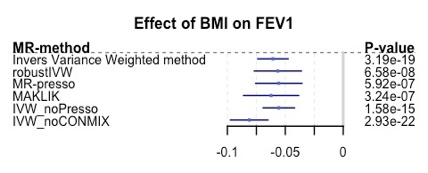

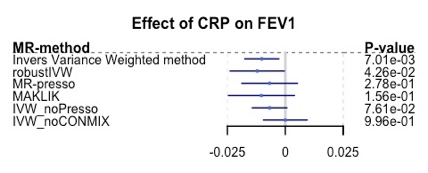

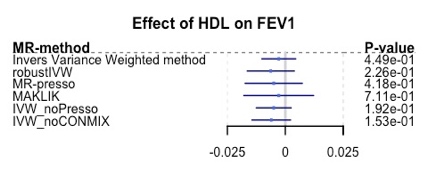

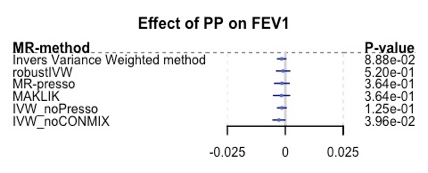

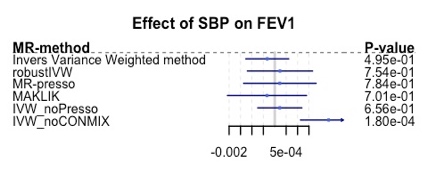

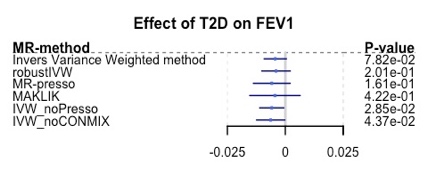

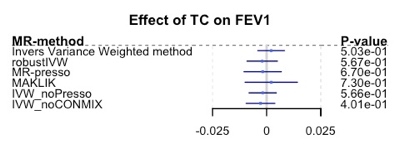

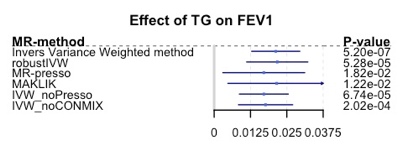

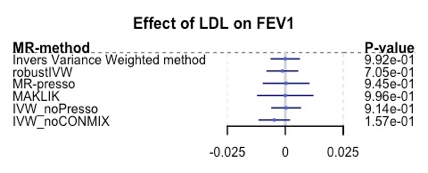

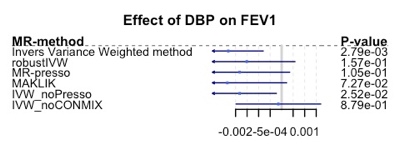


**Additional File 1 Figure S16: Sensitivity analysis: Forest plots showing effect of cardio-metabolic traits on FEV1.** Blue square represents causal estimate. Blue line is 95% confidence interval. Every line represents one approach to estimate the potential causal effect. For IVW_noPresso and IVW_noCONMIX estimates we removed variants flagged as problematic by MR Presso method or CONMIX method.


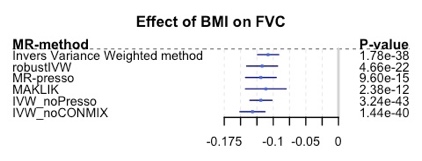

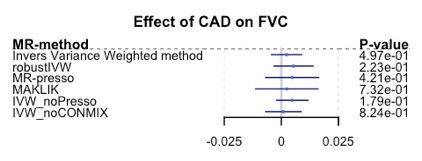

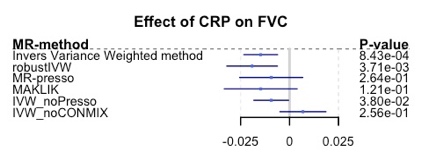

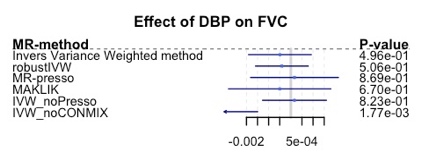

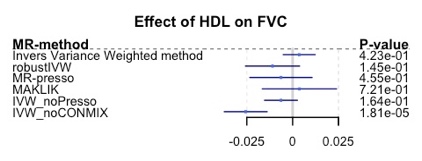

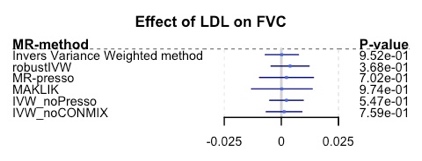

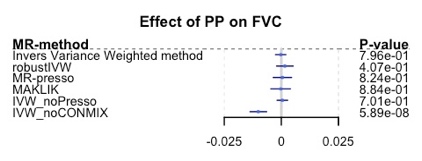

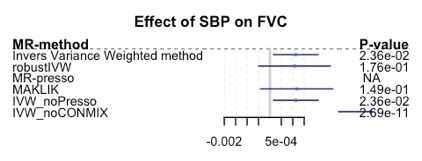

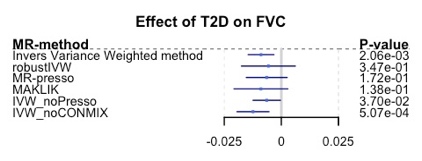

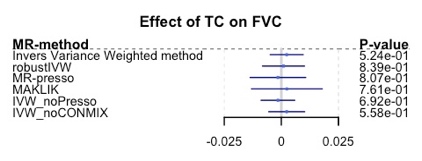

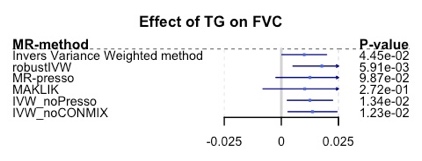


**Additional File 1 Figure S17: Sensitivity analysis: Forest plots showing effect of cardio-metabolic traits on FVC.** Blue square represents causal estimate. Blue line is 95% confidence interval. Every line represents one approach to estimate the potential causal effect. For IVW_noPresso and IVW_noCONMIX estimates we removed variants flagged as problematic by MR Presso method or CONMIX method.


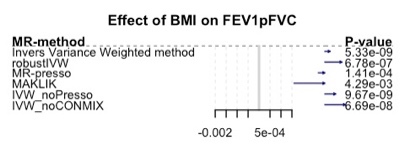

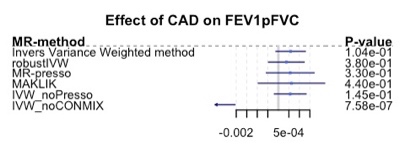

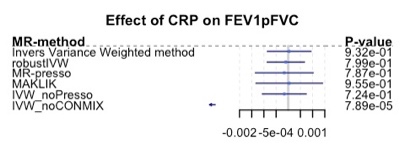

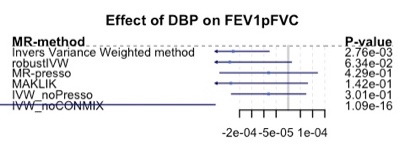

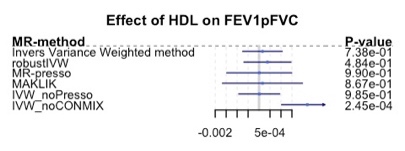

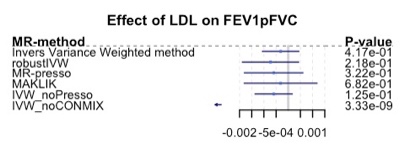

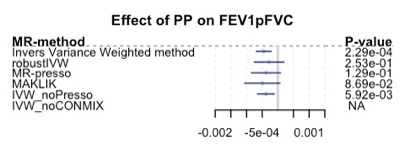

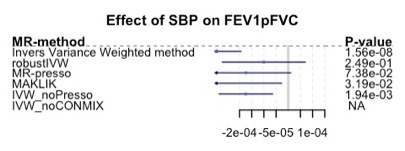

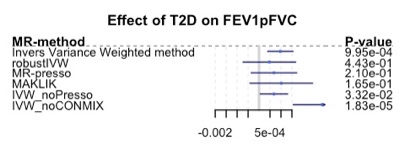

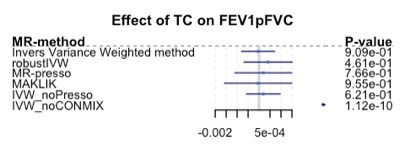

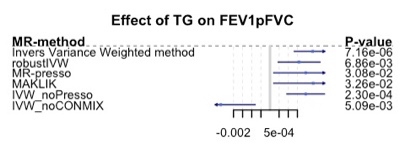


**Additional File 1 Figure S18: Sensitivity analysis: Forest plots showing effect of cardio-metabolic traits on FEV1pFVC.** Blue square represents causal estimate. Blue line is 95% confidence interval. Every line represents one approach to estimate the potential causal effect. For IVW_noPresso and IVW_noCONMIX estimates we removed variants flagged as problematic by MR Presso method or CONMIX method.


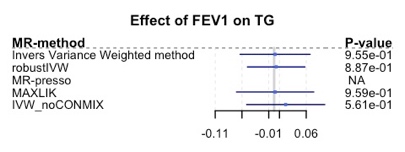

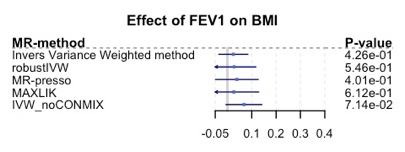

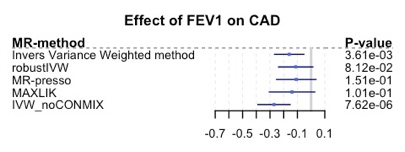

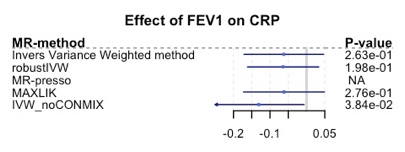

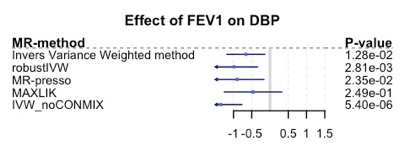

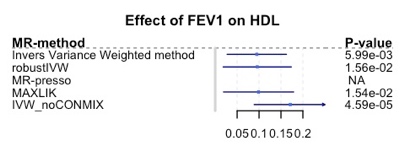

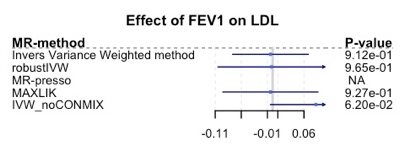

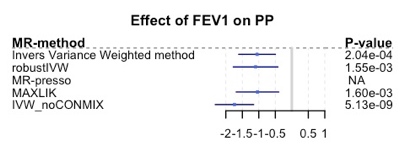

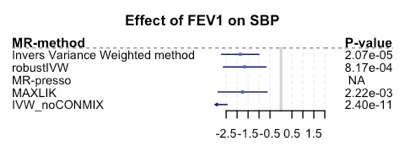

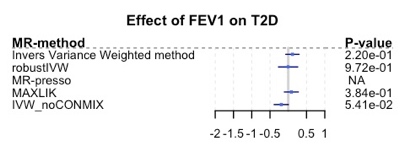

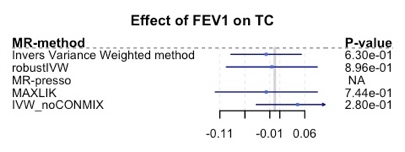


**Additional File 1 Figure S19: Sensitivity analysis: Forest plots showing effect of FEV1 on cardio-metabolic traits.** Blue square represents causal estimate. Blue line is 95% confidence interval. Every line represents one approach to estimate the potential causal effect. For IVW_noPresso and IVW_noCONMIX estimates we removed variants flagged as problematic by MR Presso method or CONMIX method.


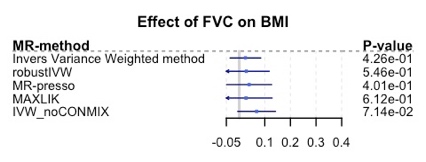

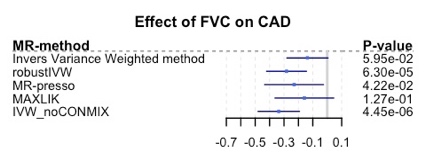

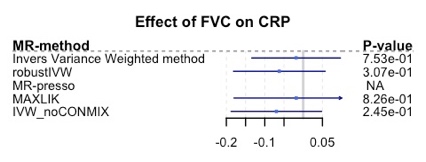

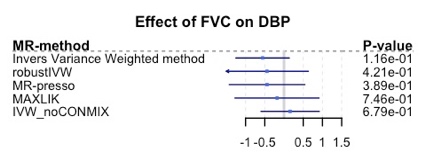

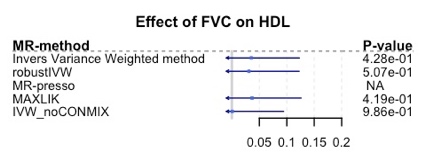

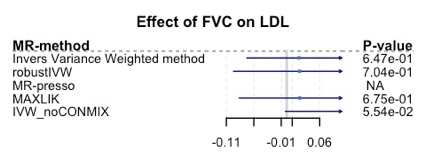

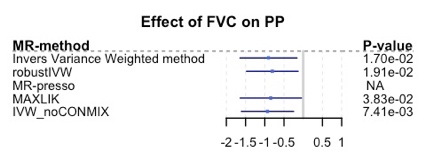

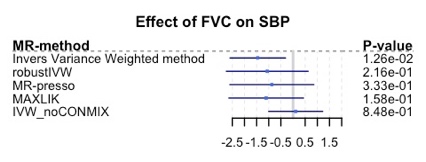

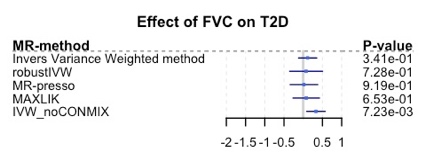

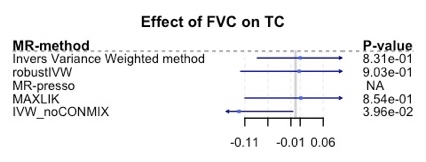

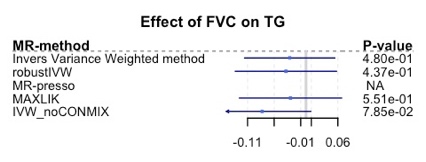


**Additional File 1 Figure S20 Sensitivity analysis: Forest plots showing effect of FVC on cardio-metabolic traits.** Blue square represents causal estimate. Blue line is 95% confidence interval. Every line represents one approach to estimate the potential causal effect. For IVW_noPresso and IVW_noCONMIX estimates we removed variants flagged as problematic by MR Presso method or CONMIX method


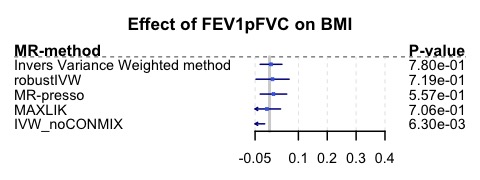

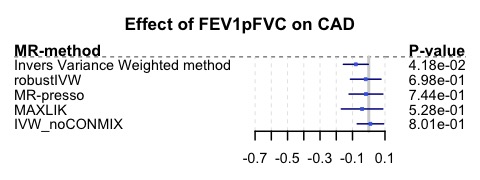

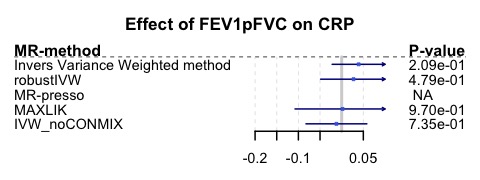

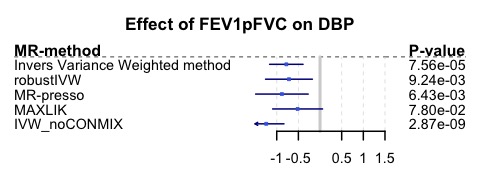

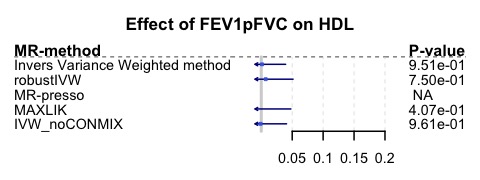

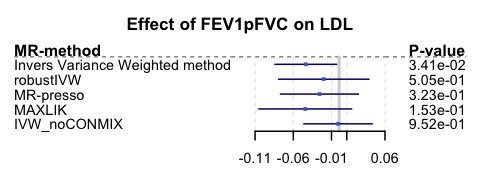

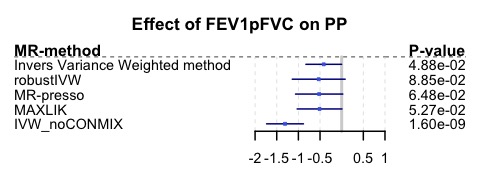

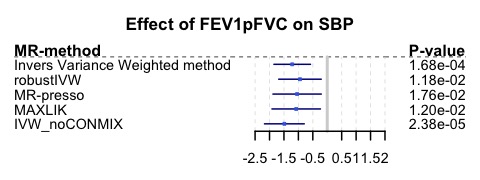

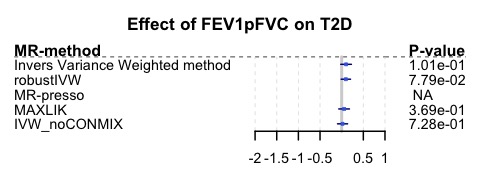

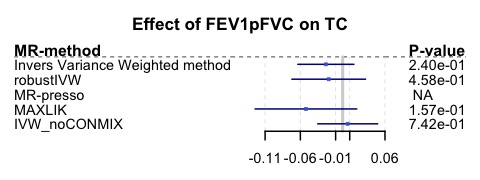

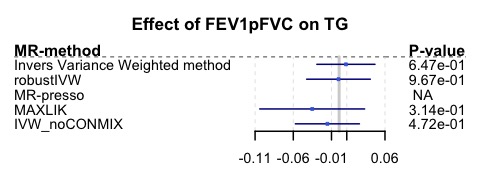


**Additional File 1 Figure S21: Sensitivity analysis: Forest plots showing effect of FEV1pFVC on cardio-metabolic traits.** Blue square represents causal estimate. Blue line is 95% confidence interval. Every line represents one approach to estimate the potential causal effect. For IVW_noPresso and IVW_noCONMIX estimates we removed variants flagged as problematic by MR Presso method or CONMIX method

**References Additional File 1**

1. Locke AE, Kahali B, Berndt SI, Justice AE, Pers TH, Day FR, et al. Genetic studies of body mass index yield new insights for obesity biology. Nature. 2015;518(7538):197-206.

2. Wain LV, Vaez A, Jansen R, Joehanes R, van der Most PJ, Erzurumluoglu AM, et al. Novel Blood Pressure Locus and Gene Discovery Using Genome-Wide Association Study and Expression Data Sets From Blood and the Kidney. Hypertension. 2017.

3. Willer CJ, Schmidt EM, Sengupta S, Peloso GM, Gustafsson S, Kanoni S, et al. Discovery and refinement of loci associated with lipid levels. Nat Genet. 2013;45(11):1274-83.

4. Nikpay M, Goel A, Won HH, Hall LM, Willenborg C, Kanoni S, et al. A comprehensive 1,000 Genomes-based genome-wide association meta-analysis of coronary artery disease. Nat Genet. 2015;47(10):1121-30.

5. Scott RA, Scott LJ, Magi R, Marullo L, Gaulton KJ, Kaakinen M, et al. An Expanded Genome-Wide Association Study of Type 2 Diabetes in Europeans. Diabetes. 2017;66(11):2888-902.

6. Dehghan A, Dupuis J, Barbalic M, Bis JC, Eiriksdottir G, Lu C, et al. Meta-analysis of genome-wide association studies in >80 000 subjects identifies multiple loci for C-reactive protein levels. Circulation. 2011;123(7):731-8.

7. Loh PR, Tucker G, Bulik-Sullivan BK, Vilhjalmsson BJ, Finucane HK, Salem RM, et al. Efficient Bayesian mixed-model analysis increases association power in large cohorts. Nat Genet. 2015;47(3):284-90.

8. Miller MR, Hankinson J, Brusasco V, Burgos F, Casaburi R, Coates A, et al. Standardisation of spirometry. Eur Respir J. 2005;26(2):319-38.

9. Bulik-Sullivan BK, Loh PR, Finucane HK, Ripke S, Yang J, Schizophrenia Working Group of the Psychiatric Genomics C, et al. LD Score regression distinguishes confounding from polygenicity in genome-wide association studies. Nat Genet. 2015;47(3):291-5.

10. Bulik-Sullivan B, Finucane HK, Anttila V, Gusev A, Day FR, Loh PR, et al. An atlas of genetic correlations across human diseases and traits. Nature genetics. 2015;47(11):1236-41.

11. Smith GD, Ebrahim S. Mendelian randomization: prospects, potentials, and limitations. Int J Epidemiol. 2004;33(1):30-42.

12. Yavorska OO, Burgess S. MendelianRandomization: an R package for performing Mendelian randomization analyses using summarized data. Int J Epidemiol. 2017;46(6):1734-9.

13. Burgess S, Butterworth A, Thompson SG. Mendelian randomization analysis with multiple genetic variants using summarized data. Genet Epidemiol. 2013;37(7):658-65.

14. Greco MF, Minelli C, Sheehan NA, Thompson JR. Detecting pleiotropy in Mendelian randomisation studies with summary data and a continuous outcome. Stat Med. 2015;34(21):2926-40.

15. Burgess S, Thompson SG. Interpreting findings from Mendelian randomization using the MR-Egger method. Eur J Epidemiol. 2017;32(5):377-89.

16. Verbanck M, Chen CY, Neale B, Do R. Detection of widespread horizontal pleiotropy in causal relationships inferred from Mendelian randomization between complex traits and diseases. Nat Genet. 2018;50(5):693-8.

17. Burgess S, Foley CN, Allara E, Staley JR, Howson JMM. A robust and efficient method for Mendelian randomization with hundreds of genetic variants. Nat Commun. 2020;11(1):376.

18. Burgess S, Thompson SG. Multivariable Mendelian randomization: the use of pleiotropic genetic variants to estimate causal effects. Am J Epidemiol. 2015;181(4):251-60.

19. Burgess S, Dudbridge F, Thompson SG. Re: "Multivariable Mendelian randomization: the use of pleiotropic genetic variants to estimate causal effects". Am J Epidemiol. 2015;181(4):290-1.

20. Wain LV, Shrine N, Artigas MS, Erzurumluoglu AM, Noyvert B, Bossini-Castillo L, et al. Genome-wide association analyses for lung function and chronic obstructive pulmonary disease identify new loci and potential druggable targets. Nat Genet. 2017;49(3):416-25.

21. Burgess S. Sample size and power calculations in Mendelian randomization with a single instrumental variable and a binary outcome. Int J Epidemiol. 2014;43(3):922-9.

22. Brion MJ, Shakhbazov K, Visscher PM. Calculating statistical power in Mendelian randomization studies. Int J Epidemiol. 2013;42(5):1497-501.
